# The Therapeutic Landscape of Rheumatoid Arthritis: Current State and Future Directions

**DOI:** 10.3389/fphar.2021.680043

**Published:** 2021-05-28

**Authors:** Shahin Shams, Joseph M. Martinez, John R. D. Dawson, Juan Flores, Marina Gabriel, Gustavo Garcia, Amanda Guevara, Kaitlin Murray, Noah Pacifici, Maxemiliano V. Vargas, Taylor Voelker, Johannes W. Hell, Judith F. Ashouri

**Affiliations:** ^1^ Department of Biomedical Engineering, University of California, Davis, Davis, CA, United States; ^2^ Department of Pharmacology, University of California, Davis, Davis, CA, United States; ^3^ Department of Physiology and Membrane Biology, University of California, Davis, Davis, CA, United States; ^4^ Center for Neuroscience, University of California, Davis, Davis, CA, United States; ^5^ Department of Anatomy, Physiology, and Cell Biology, University of California, Davis, Davis, CA, United States; ^6^ Department of Chemistry, University of California, Davis, Davis, CA, United States; ^7^ Rosalind Russell and Ephraim R. Engleman Rheumatology Research Center, Department of Medicine, University of California, San Francisco, CA, United States

**Keywords:** rheumatoid arthritis, autoimmune disease, inflammatory cytokines and chemokines, adenosine receptor, JAK-STAT signaling, biological therapies, disease modifying anti-rheumatic drugs, nanoparticles

## Abstract

Rheumatoid arthritis (RA) is a debilitating autoimmune disease with grave physical, emotional and socioeconomic consequences. Despite advances in targeted biologic and pharmacologic interventions that have recently come to market, many patients with RA continue to have inadequate response to therapies, or intolerable side effects, with resultant progression of their disease. In this review, we detail multiple biomolecular pathways involved in RA disease pathogenesis to elucidate and highlight pathways that have been therapeutic targets in managing this systemic autoimmune disease. Here we present an up-to-date accounting of both emerging and approved pharmacological treatments for RA, detailing their discovery, mechanisms of action, efficacy, and limitations. Finally, we turn to the emerging fields of bioengineering and cell therapy to illuminate possible future targeted therapeutic options that combine material and biological sciences for localized therapeutic action with the potential to greatly reduce side effects seen in systemically applied treatment modalities.

## Introduction

Rheumatoid Arthritis (RA) is a chronic, destructive autoimmune disease that afflicts over one percent of the world population and causes substantial pain, joint deformity, and functional disability (Helmick et al., 2008). It is characterized by inflammation of the synovial membrane lining joints, frequently resulting in bone erosion and eventual joint destruction if left untreated. It can also affect extra-articular organs (e.g., heart, lungs, eyes, blood vessels) and reduce life span (Hakala, 1988; Young and Koduri, 2007; Koduri et al., 2010; Widdifield et al., 2018). Additionally, autoantibodies to rheumatoid factor (RF) and citrullinated protein are often present. Risk factors for RA include smoking, gender (females show higher incidence), obesity, old age, and genetics with genetic and epigenetic factors comprising ∼30% of risk (reviewed in (Ollier and MacGregor, 1995; Scott et al., 2010; Smolen et al., 2018; Mikhaylenko et al., 2020; Smolen et al., 2020)). In North America, the overall prevalence of RA is ∼1% (Myasoedova et al., 2010; Tobón et al., 2010) though some groups show higher prevalence rates – with the highest prevalence affecting the Chippewa Native American people at 7% (Alamanos and Drosos, 2005; Ferucci et al., 2005). The yearly cost of care for the chronic treatment of RA in the United States is estimated at $12,509 (direct treatments costs of $3,725) in patients using non-biologic treatments, and $36,053 (direct treatment costs of $20,262) in patients using biologic agents (Hresko et al., 2018). It has been suggested that these high treatment costs may negatively affect medication adherence in patients with RA (Heidari et al., 2018).

Final common mediators of disease, including tumor necrosis factor-α (TNF-α) and interleukin (IL)-6, are well studied and have yielded breakthrough therapeutics. Although therapeutic options are increasing, many patients continue to have an inadequate response to therapy or intolerable side effects (Alonso-Ruiz et al., 2008; Wang et al., 2018). In this review we will discuss currently available and emerging treatments, as well as their described mechanisms of action (Table 1). We will also propose and explore potential novel therapeutic strategies for future drug development for the treatment of RA.

**TABLE 1:** FDA approved drugs to treat RA.

Name	Drug Type	Drug Class	Year Approved for RA	Molecular Weight	Chemical Structure	References
Methotrexate	Small molecule	Antimetabolite	1988	454.45 g/mol	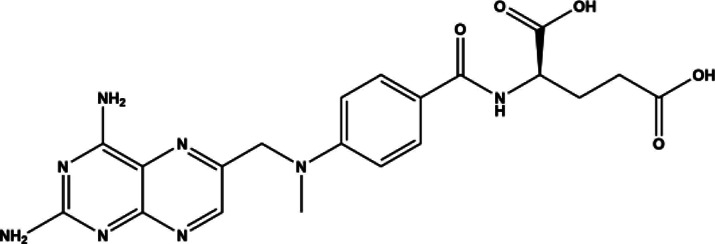	Smolen et al. (2020); Malaviya (2016); Gubner et al. (1951); Wright et al. (1951); Rajagopalan et al. (2002); Hertz et al. (1956); Pannu (2019); Weinblatt et al. (1985); Tian and Cronstein (2007); Vega (2015); Brown et al. (2016); Wessels et al. (2008); Cronstein (2005); Yamamoto et al. (2016); Cronstein and Aune (2020); Nesher and Moore (1990); Nesher et al. (1996); Lawson et al. (2007); Chan and Cronstein (2010); Cronstein et al. (1991); Baggott et al. (1986); Cronstein et al. (1993); Morabito et al. (1998); Prabhakar et al.and (1995); Cronstein et al. (1986); Attar (2010); Hamid et al. (2018); Shiroky et al. (1993); Lindsay et al. (2009); Provenzano (2003); Albrecht and Müller-Ladner (2010); Bathon et al. (2000); Goekoop-Ruiterman et al. (2007); van Vollenhoven et al. (2009); Emery et al. (2012); Weinblatt et al. (1994); Singh et al. (2015); Breedveld et al. (2006); Heo et al. (2017); Matera et al. (2018)
Sulfasalazine	Compound molecule	Anti-inflammatory	1950	398.39 g/mol	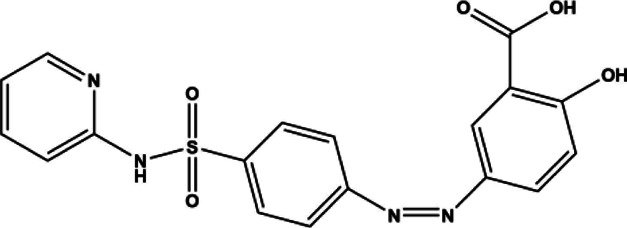	Morabito et al. (1998); Choi and Fenando (2020); O'Dell (1998); Wahl et al. (1998); Cronstein et al. (1999); Park et al. (2019); Rodenburg et al. (2000); Lee et al. (2004); Hirohata et al. (2002); Volin et al. (2002); Suarez-Almazor et al. (2000b); O'Dell et al. (2002); Moreland et al. (2012); Curtis et al. (2020); Erhardt et al. (2019)
Hydroxychloroquine	Small molecule	Antimalarial	1956	433.95 g/mol	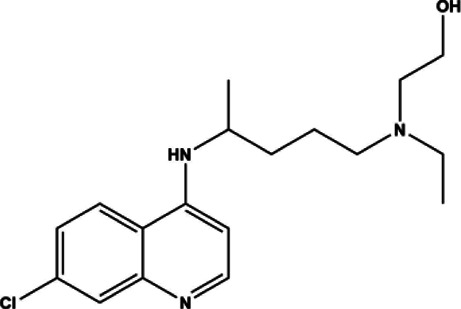	(Singh et al. (2015); O'Dell et al. (1996); Moreland et al. (2012); Administration USFD (2020); Schrezenmeier and Dorner (2020); Mok et al. (2005); Rempenault et al. (2018); Ruiz-Irastorza et al. (2009); Sharma et al. (2016); Tsakonas et al. (2000); Grigor et al. (2004); Circu et al. (2017); Mauthe et al. (2018); Wu et al. (2017); Rebecca et al. (2019); Ewald et al. (2008); Kuznik et al. (2011); Hjorton et al. (2018); Wallace et al. (1994); Wallace et al. (1993); Suarez-Almazor et al. (2000a); Koffeman et al. (2009); Ravindran and Alias (2017); Carmichael et al. (2002); Tett et al. (1989); Trnavský et al. (1993); Ma and Xu (2013)
Prednisone	Small molecule	Corticosteroid	2012	358.43 g/mol	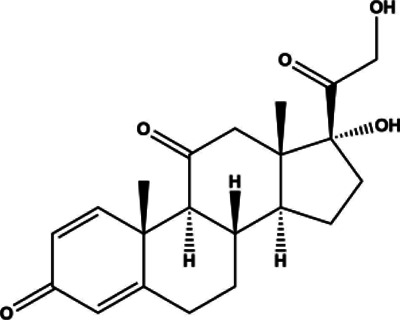	van Everdingen et al. (2002); Wassenberg et al. (2005); Jacobs et al. (2006); Malysheva et al. (2008); Hafstrom et al. (2009); Pincus et al. (2009); Bakker et al. (2012); Krasselt and Baerwald (2014)
Tofacitinib	Small molecule	JAK inhibitor	2012	312.37 g/mol	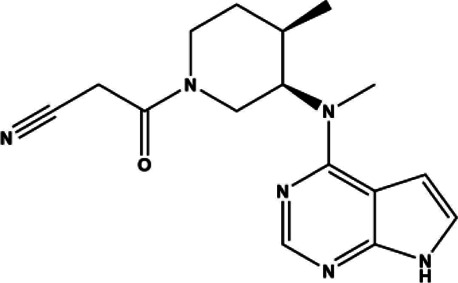	Ghoreschi et al. (2011); Kwok (2014); Hodge et al. (2016); Schwartz et al. (2016); Keystone et al. (2017); Grigoropoulos et al. (2019); Itamiya et al. (2020)
Baricitinib	Small molecule	JAK inhibitor	2018	371.42 g/mol	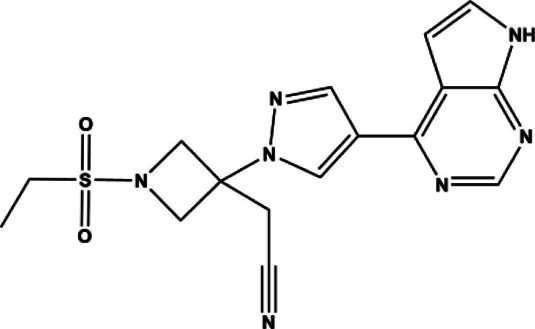	(Ghoreschi et al., 2011; Keystone et al., 2017)
Upadacitinib	Small molecule	JAK inhibitor	2019	398.38 g/mol	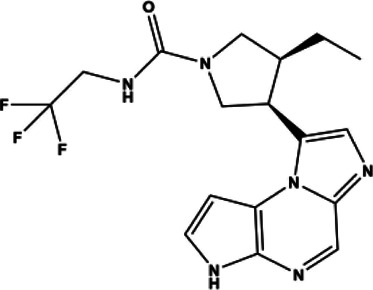	Genovese et al. (2018); Parmentier et al. (2018); Brooks (2019)
Anakinra	Biologic	Interleukin antagonist	2001	17.3 kDa	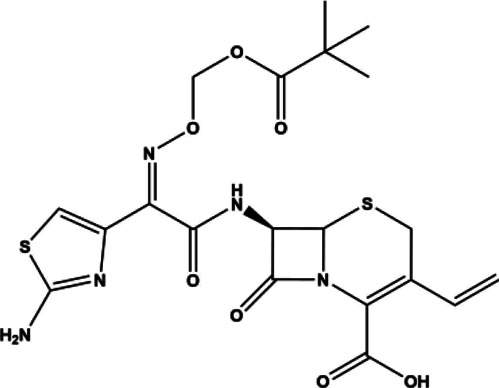	Arend et al. (1990); Bresnihan et al. (1998); Cohen et al. (2002); Buch et al. (2004); Genovese et al. (2004); Mertens and Singh (2009a); Mertens and Singh (2009b); England et al. (2018)
Etanercept	Biologic	TNF inhibitor	1998	150 kDa	Humanized monoclonal antibody fragment fusion protein	Bathon et al. (2000); Genovese et al. (2004); Hetland et al. (2010); Emery et al. (2012); Moreland et al. (2012); Machado et al. (2013)
Abatacept	Biologic	TNF inhibitor	2005	92 kDa	Fully humanized monoclonal antibody	Kremer et al. (2006); Maxwell and Singh (2009); Blair and Deeks (2017)
Infliximab	Biologic	TNF inhibitor	1999	149.1 kDa	Chimeric (murine/human) monoclonal antibody	Lisman et al. (2002); Perdriger (2009); van Vollenhoven et al. (2009); Vermeire et al. (2009); Hetland et al. (2010); Monaco et al. (2015); Braun and Kay (2017); Scherlinger et al. (2017)
Adalimumab	Biologic	TNF inhibitor	2002	148 kDa	Fully humanized monoclonal antibody	Breedveld et al. (2006); Machado et al. (2013); Burmester et al. (2017); Keystone et al. (2017)
Golimumab	Biologic	TNF inhibitor	2009	150 kDa	Fully humanized monoclonal antibody	Braun and Kay (2017)
Certolizumab	Biologic	TNF inhibitor	2009	91 kDa	Humanized monoclonal antibody fragment conjugated to a PEG moiety	Kaushik and Moots (2005); Goel and Stephens (2010); Choy et al. (2012)
Tocilizumab	Biologic	IL-6 receptor inhibitor	2010	148 kDa	Humanized monoclonal antibody	Jones et al. (2010); Fleischmann et al. (2013); Kaneko (2013); Lee et al. (2014); Gale et al. (2019)
Sarilumab	Biologic	IL-6 receptor inhibitor	2017	150 kDa	Fully humanized monoclonal antibody	Tanaka and Martin Mola (2014); Burmester et al. (2017); McCarty and Robinson (2018)
Rituximab	Biologic	Anti-CD20	2006	145 kDa	Chimeric (murine/human) monoclonal antibody	Reff et al. (1994); Anderson et al. (1997); Clynes et al. (2000); Edwards and Cambridge (2001); Shaw et al. (2003); Edwards et al. (2004); Weiner (2010); Keystone et al. (2012)

### Pharmacology of Methotrexate and Use in RA

Prior to the identification of methotrexate (MTX), options for the treatment of RA were quite limited. Treatments for RA in the early twentieth century predominantly focused on gold therapy, in which gold salts were applied via either injection or oral administration (Davis, 1988; Clark et al., 2000). In the mid-twentieth century, another potential power player, penicillamine, a derivative of penicillin, was first demonstrated to improve RA disease activity compared to placebo (Suarez-Almazor et al., 2000c). Though these therapeutic options demonstrated efficacy in treating RA, they were also plagued with serious incidents of toxicity (Clark et al., 2000; Suarez-Almazor et al., 2000c). These treatment modalities fell out of favor over time with the identification and application of small molecule compounds that could improve RA disease activity with less toxicity. One of the most impactful of these compounds was MTX.

MTX is a small organic antimetabolite used as a chemotherapy agent and immune system suppressant (Table 1). Despite advancements in new therapeutics, it continues to be the first-line therapy and standard of care for the treatment of RA. First developed in 1947 by a team of researchers led by Sidney Farber, MTX was initially used as a chemotherapeutic in the treatment of childhood leukemia. Farber and his colleagues made the observation that administering folic acid to tumor-carrying mice made the tumors proliferate (Malaviya, 2016). Farber’s group reasoned that if folic acid worsened tumor growth then depriving tumors of folic acid could prevent cellular proliferation. A team of chemists at the Lederle pharmaceutical company lead by Yella Subbarow synthesized a folic acid analogue, aminopterin. This analogue prevented folic acid from being metabolized and used in DNA synthesis, thus arresting tumor growth. However, due to a lack of stability and a complex synthesis, in 1950 aminopterin was replaced by amethopterin, another antimetabolic analogue of folic acid, now known as MTX.

In 1951 Gubner and colleagues (Gubner et al., 1951) demonstrated that MTX had anti-cancer properties, causing remission in breast cancer (Wright et al., 1951). MTX exerts its effect by binding and inhibiting dihydrofolate reductase (K_D_ of 9.5 nM), an enzyme that is critical for the synthesis of the anabolic cofactor tetrahydrofolic acid (Rajagopalan et al., 2002). This was the first study to show it had efficacy in solid tumors, expanding its use. In 1956 MTX cured metastatic cancer, the first therapeutic to achieve this feat (Hertz et al., 1956). Due to its low cost of production, relative safety, and efficacy MTX continues to be one of the most prescribed medicines in the United States (Pannu, 2019).

It was later discovered by Gubner and colleagues that at low doses, MTX has “steroid-like” effects and could be used for a wide array of diseases, including psoriatic arthritis and RA (Weinblatt et al., 1985). One of the initial observations with aminopterin was the inhibition of connective tissue proliferation. This observation led to a study in 1951 by Gubner et al. in RA (Gubner et al., 1951). The results of this study showed that it caused rapid improvement in RA signs and symptoms in the majority of patients. This initial discovery triggered the development of MTX as a first-line treatment of RA. In low doses it serves as a potent immune system suppressant and has anti-inflammatory properties. By 1985 it was clinically demonstrated to be a potent and effective treatment for RA (Tian and Cronstein, 2007); patients treated with MTX are more likely to reach ACR50 in their RA disease score compared to placebo on the American College of Rheumatology scale, which signifies both a 50% improvement in the number of tender and swollen joints and a 50% improvement in at least 3 of 5 disease assessment criteria (ACR20, ACR50 and ACR70 being commonly used assessment scores) (Vega, 2015).

Today, MTX is used as one of the first interventions in patients with RA, with weekly dosages ranging from 5 to 25 mg (Vega, 2015), though therapeutic doses range closer to 15–25 mg weekly. MTX is inexpensive compared to newer biologic drugs. In addition, it effectively treats erosive RA (Brown et al., 2016). Thus, it is commonly the first therapeutic prescribed for RA. In fact, the European League Against Rheumatism recommend that if no contraindications exist, newly diagnosed RA patients be treated with MTX and glucocorticoids for at least three months while monitoring for improvement before proceeding to treatment with biologics (Smolen et al., 2020).

There are multiple mechanisms of action (MOA) for MTX. Below, we address MTX’s ability to 1) suppress lymphocyte proliferation via inhibition of purine and pyrimidine synthesis, 2) suppress transmethylation reactions thus diminishing accumulation of polyamines, and 3) induce adenosine mediated suppression of inflammation (Wessels et al., 2008). It is currently unknown which MOA is primarily responsible for its efficacy in treating RA and is more likely a combination of these mechanisms.

**FIGURE 1:**
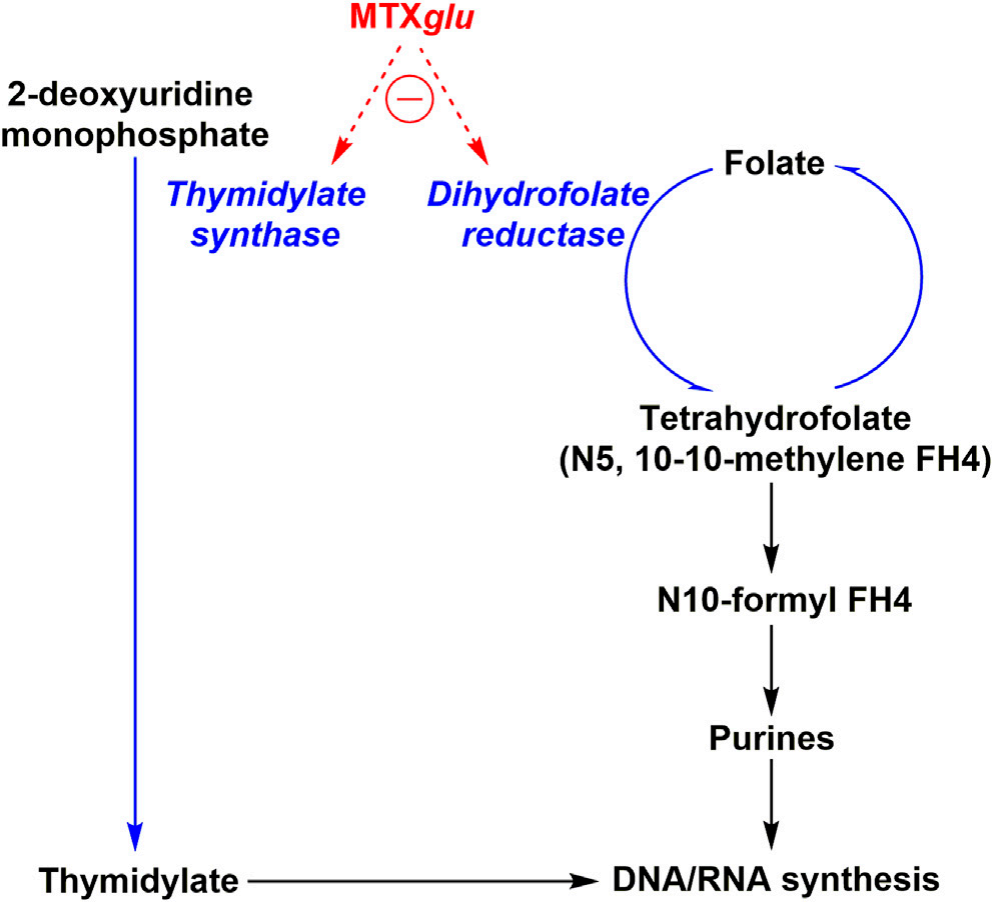
MTX toxicity mechanism of action. Oligonucleotide synthesis is suppressed two-fold by MTX*glu* (methotrexate polyglutamate) via thymidylate synthase and dihydrofolate reductase inhibition.

The best-known MOA of MTX is its action as a competitive antagonist of dihydrofolate reductase (DHFR), an enzyme that participates in tetrahydrofolate (THF) synthesis as shown in Figure 1. MTX is taken up by cells via the transmembrane protein reduced folate carrier 1 (RFC1) and is quickly polyglutamylated by folylpolyglutamate synthase (FPGs) to MTX_Glu_ – a bioactive metabolite which is stable for a period of weeks, thus allowing for continued low dose administration to result in accumulation in target tissues (Cronstein, 2005; Yamamoto et al., 2016; Cronstein and Aune, 2020). MTX_Glu_ is a highly potent inhibitor of many enzymes, including DHFR (Cronstein and Aune, 2020). MTX, a structural analog of folate, competitively inhibits DHFR by binding to the enzymatic site of action. Inhibition of DHFR inhibits tetrahydrobiopterin (BH4) production, and thus inhibits nitric oxide (NO) production – thereby increasing the presence of intracellular reactive oxygen species (ROS), activating JUN N-terminal kinase (JNK) which regulates apoptotic sensitivity and cell cycle progression in an anti-inflammatory context (Cronstein and Aune, 2020). DHFR also inhibits NF-κB translocation to the nucleus in a JNK dependent manner, although the exact mechanism remains to be elucidated. THF is necessary to produce purines and as a cofactor for thymidylate synthetase by donating a methyl group. Thymidylate as well as purines are necessary for DNA and RNA synthesis. This aspect allows MTX to serve as an effective chemotherapy agent; reducing DNA/RNA synthesis has a dramatic hampering effect on the ability for rapidly dividing host cells, like cancer cells, to proliferate. DNA/RNA synthesis inhibition is also what is largely responsible for MTX induced toxicities. Low-dose MTX was believed to alleviate RA symptoms by decreasing proliferation of lymphocytes that are responsible for causing inflammation of the synovial joint. However, it was unclear, whether this was the sole MOA as low-dose MTX is only taken once a week, thus potentially only providing short term inhibition of lymphocyte mediated inflammation. This led to the exploration of additional MOAs that could also be involved.

Polyamine accumulation has been observed in synovial fluids, urine, and mononuclear cells in patients with RA (Cronstein and Aune, 2020). These polyamines, including spermine and spermidine, are hydrolyzed to hydrogen peroxide and ammonia by monocytes – which act as cytotoxins that damage joint tissues (Nesher and Moore, 1990; Nesher et al., 1991; Nesher et al., 1996). It was hypothesized that MTX prevented the inflammatory and cytotoxic function of immune cells in the joints of patients with RA by inhibiting transmethylation and thereby suppressing polyamine accumulation in joints and other tissues (Cronstein and Aune, 2020). Though transmethylation and its role in inflammatory pathway activation is well documented (Lawson et al., 2007; Cronstein and Aune, 2020), inhibition of transmethylation alone failed to improve the clinical course of RA (Chan and Cronstein, 2010). This latter finding suggests that the inhibition of transmethylation reactions likely plays a small part in MTX’s anti-inflammatory effects in RA and potentially other related inflammatory diseases.

**FIGURE 2:**
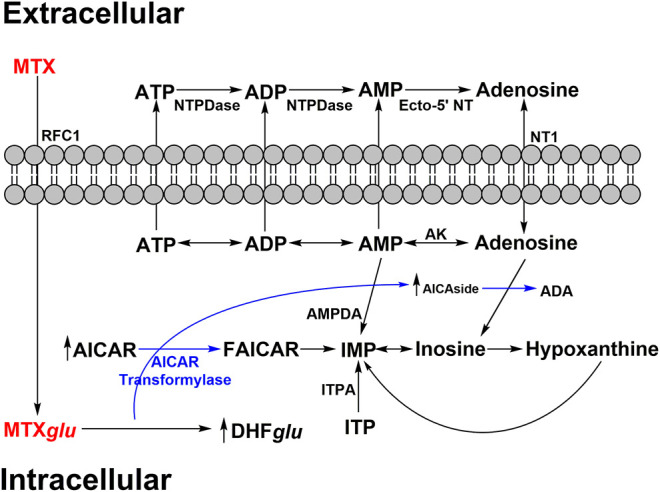
MTX impact on adenosine secretion. MTX is polyglutamylated (MTX*glu*) after active transport of MXT into intracellular space. MTX*glu* inhibits AMP/adenosine deaminase (AMPDA/ADA respectively) and thus IMP/inosine production through accumulation of aminoimidazole carboxamidoribonucleotide (AICAR) and aminoimidazole carboxamidoribonucleoside (AICAside), the intermediate metabolites of purine biosynthesis. This results in increased cellular release of adenine nucleotides which are quickly converted into adenosine in the extracellular space. Adenosine triphosphate – ATP; adenosine diphosphate – ADP; adenosine monophosphate – AMP; adenylate deaminase – AMPDA; dihydrofolate polyglutamate - DHF*glu*; formyl AICAR - FAICAR; Inosine monophosphate – IMP; inosine triphosphate – ITP; inosine triphosphate pyrophosphatase – ITPA; reverse folate carrier 1 – RFC1; adenosine kinase – AK; nucleoside triphosphate phosphohydrolase – NTPDase; ecto-5’ nucleotidase – Ecto-5’ NT.

MTX is also known to increase extracellular adenosine release (Cronstein et al., 1991) as shown in Figure 2. MTX and its metabolites are taken up by cells via reduced folate carriers where they subsequently undergo polyglutamylation, to form MTX_Glu_, a biologically active metabolite that can persist and build up in cells for extended periods of time. This aspect explains why RA patients typically only require a low dose once a week (Cronstein, 2005). MTX_Glu_ is a potent inhibitor of 5-aminoimidazole-4-carboxamide ribonucleotide (AICAR) transformylase. Inhibition leads to a buildup of AICAR over time (Baggott et al., 1986). Accumulation of AICAR leads to the inhibition of adenosine monophosphate (AMP) deaminase as well as adenosine deaminase. This blocks the conversion of AMP to inosine monophosphate (IMP) and adenosine to inosine, respectively. The buildup of intracellular AMP and adenosine promotes release of adenosine metabolites via an unidentified mechanism (Cronstein et al., 1993). The equilibrative nucleoside transporter 1 (ENT1) is believed to play a role in the increase in extracellular adenosine. It has since been demonstrated that the MTX mediated increases in extracellular adenosine are generated extracellularly via ecto-5’-nucleotidase, an enzyme that converts AMP to adenosine (Morabito et al., 1998).

**FIGURE 3:**
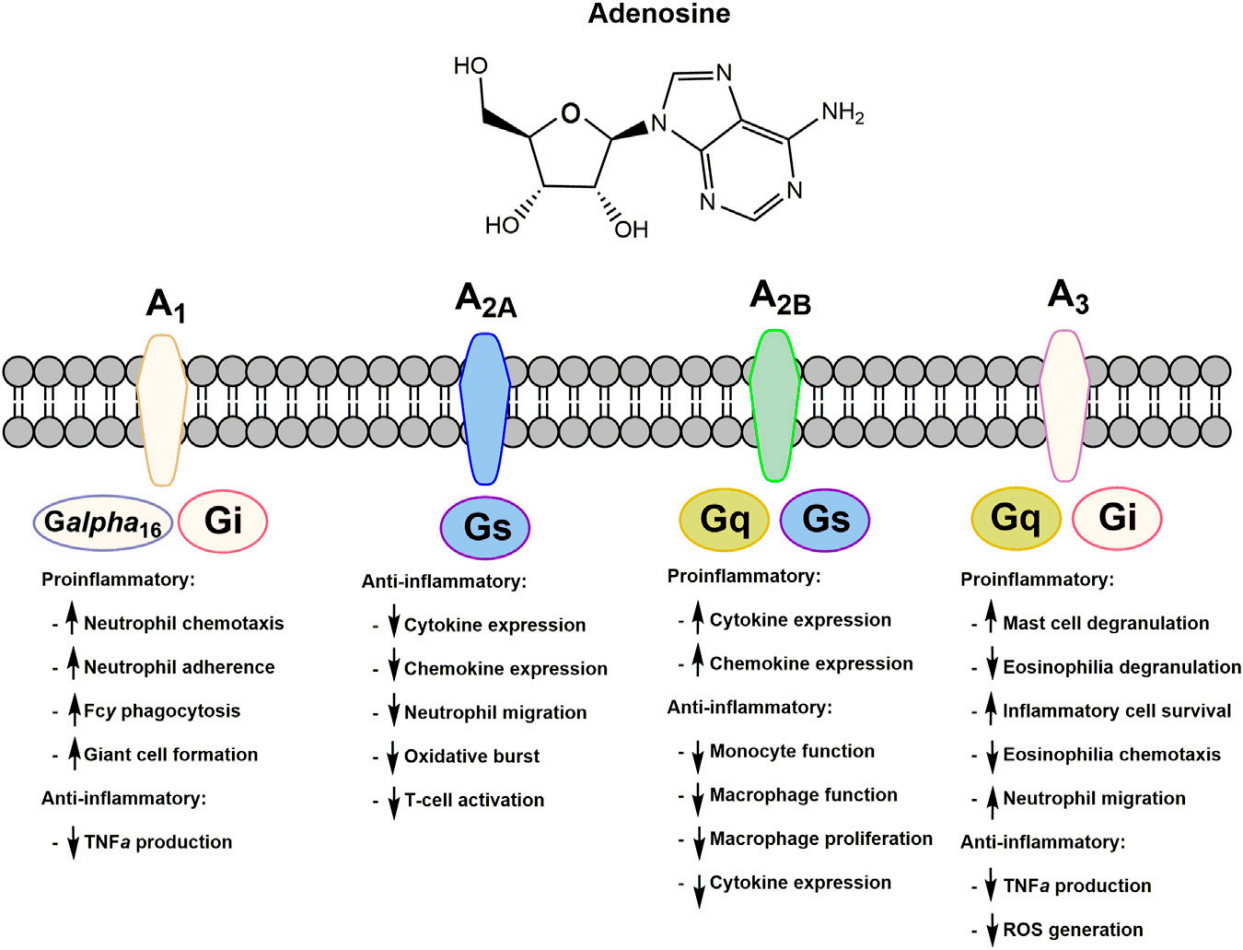
Adenosine receptors and their respective proinflammatory and anti-inflammatory responses upon extracellular adenosine binding. All adenosine receptors are a part of the G-protein coupled receptor family. Respective G-protein signaling partners are indicated on each subtype of adenosine receptor.

Extracellular adenosine binds to specific adenosine G-protein coupled receptors (GPCRs) as summarized in Figure 3. Four distinct subtypes are known, A_1_, A_2_, A_2B_, and A_3_, which have demonstrated a variety of both proinflammatory and anti-inflammatory responses (Blackburn et al., 2009). Adenosine can have anti-inflammatory effects mediated through a combination of adenosine receptor activation. For instance, it can inhibit the production of anti-TNF-α, although the adenosine receptor(s) involved in this action remains controversial (Prabhakar et al., 1995). Adenosine also inhibits adherence to endothelial cells by stimulated neutrophils, an important event that guides neutrophil recruitment into an inflammatory site through adhesion to the vascular endothelium (Cronstein et al., 1986). Decreasing the recruitment of neutrophils to the endothelial cells at the site of inflammation can decrease the production of inflammatory cytokines. Activation of the A_2_ receptor is known to inhibit neutrophil oxidative activity and protects endothelial cells from neutrophil mediated injury. The contribution of specific adenosine receptor subtypes in various cell types is complex and the mechanisms involved in the regulation of inflammation are not completely understood. However, data support the hypothesis that activation of adenosine receptors, due to increases in extracellular adenosine, is primarily responsible for mediating the anti-inflammatory effect of MTX and allowing it to serve as an effective treatment for RA.

MTX has its share of side effects although it is generally well tolerated and overall has a good safety profile. Side effects are dose dependent and thus RA doses do not tend to induce the same degree of side effects as doses used for chemotherapy. Most side effects arise due to deficits in folic acid metabolism. Toxicities from low-dose MTX related to decreases in folic acid metabolism include anemia, neutropenia, stomatitis, and oral ulcers (Attar, 2010; Hamid et al., 2018). These can generally be prevented or alleviated by folate supplementation (Shiroky et al., 1993). Toxicities unrelated to suppression of folate metabolism include hepatic fibrosis (Lindsay et al., 2009), pulmonary fibrosis (Provenzano, 2003), lethargy, fatigue, renal insufficiency, and rarely accelerated nodulosis (Albrecht and Müller-Ladner, 2010). MTX is also a teratogen and contraindicated during pregnancy and breast-feeding, as well as for men and women in the months preceding conception.

MTX is no less efficacious than specific anti-TNF therapy for the relief of symptoms including joint inflammation in early RA when long-term outcomes are examined (Bathon et al., 2000). Approximately 1/3 of patients will have a dramatic therapeutic response with MTX monotherapy and may not require any additional treatments (Goekoop-Ruiterman et al., 2007; van Vollenhoven et al., 2009; Emery et al., 2012). MTX also has a favorable adherence rate. In a 5-year prospective study 64% of patients completed the 5-year study, and only 7% withdrew due to lack of efficacy. A significant sustained clinical response, improvement in functional status, and a reduction in sedimentation rate was observed (Weinblatt et al., 1994). However, for a majority of patients, MTX monotherapy is insufficient to fully control their RA disease activity. For these patients, the addition of other conventional synthetic disease modifying anti-rheumatic drugs (csDMARDs), such as sulfasalazine and hydroxychloroquine, biological DMARDs (bDMARDs) or alternative treatments are added or used in lieu of MTX. In summary, while the exact anti-inflammatory MOA has yet to be elucidated, and despite its range of toxicities, MTX remains the cornerstone for RA therapy. Due to its low cost and efficacy, the American College of Rheumatology recommends MTX as the initial and first-line therapy to treat RA (Singh et al., 2015). It will likely continue to serve as an effective initial treatment strategy for RA and in addition to biologics to manage RA, particularly as MTX monotherapy has been shown to outperform at least one biologic as monotherapy (Breedveld et al., 2006).

It would be ideal if one could harness MTX’s efficacy in RA without its side effects through a more localized administration. Unfortunately, no such treatment currently exists. However, there are some promising recent studies which may provide insights into the targeted administration of MTX.

One of these studies comes from Sungkyunkwan University, where investigators administered MTX-loaded dextran sulfate (DS-MTX) nanoparticles to mice with collagen-induced arthritis (CIA) as a model for RA (Heo et al., 2017). After intravenous injection of the DS-MTX nanoparticles, they used near-infrared fluorescence PET to visualize their localization. They found that the DS-MTX nanoparticles were selectively taken up by activated macrophages and significantly enriched in the inflamed joints of the arthritic mice compared to non-arthritic wildtype controls. Additionally, they observed that CIA mice treated with DS-MTX significantly reduced cartilage erosion and synovial inflammation compared to CIA mice that received free MTX intravenously. This suggests that a more directed delivery of MTX treatment using DS-MTX nanoparticles could provide improved efficacy compared to more traditional methods of administration. Although this approach is early in the development process, it could prove to be a promising delivery system for the treatment of RA, as well as other organ-specific autoimmune diseases.

A group from Spain has found another creative way to administer MTX to RA patients in the hopes of limiting side effects. Carlo Matera and colleagues describe a photoactivatable derivative of MTX which they have named phototrexate (Matera et al., 2018). Phototrexate has a double bond which can adopt a therapeutically active cis conformation upon activation by UV-light and relaxes to an inactive trans conformation in the absence of light. The study suggests that cis phototrexate has an affinity similar to MTX for dihydrofolate reductase with an IC_50_ of 6 nM. The trans isomer phototrexate has a significantly reduced efficacy with an IC_50_ of 34 µM. Thus, administration of phototrexate followed by photoactivation could provide a new treatment modality not only for RA, but also in cancer, with the ability to spatiotemporally control the activity, and thereby the toxicity, of the drug. It is important to note that the light wavelength necessary to activate this small molecule exhibits low skin penetration and therefore this iteration serves more as a proof of concept for localized therapeutic options for RA (Matera et al., 2018). Further development of photoactivatable drugs activated by wavelengths that penetrate the body orders of magnitude better (such as those near the infrared region) will greatly expand the potential clinical impact of this technology (Matera et al., 2018).

### Sulfasalazine in RA

#### Overview

Sulfasalazine (SSZ) is a csDMARD FDA approved to manage several rheumatic diseases including polyarticular juvenile idiopathic arthritis, ulcerative colitis, and RA. First approved by the FDA for medical use in 1950, SSZ has been used for decades either alone or in combination with other RA therapeutics for treating rheumatic diseases. SSZ is a prodrug consisting of 5-aminosalicyclic acid and sulfapyridine linked via an azo bond which is cleaved via bacteria located in the colon, releasing the active compound 5-aminosalicyclic acid (Choi and Fenando, 2020). Though effective, inexpensive, easy to administer, and not known to impact fetal development, SSZ is associated with side effects including nausea, vomiting, anorexia, headache, and skin rash, as well as several adverse events including blood dyscrasias, pancreatitis, interstitial nephritis, hepatitis, and hepatic failure. Therefore, close and frequent monitoring of liver function tests, complete blood count, and serum creatinine in the first 3 months is very important, followed by every 8 to 12 thereafter (Ransford and Langman, 2002; Choi and Fenando, 2020). SSZ is no longer frequently utilized as a monotherapy, but is commonly used for management of RA as a part of the classic triple therapy regimen alongside hydroxychloroquine (HCQ) and MTX (O'Dell, 1998). SSZ is administered orally twice a day in 500 mg tablets and is available in both immediate and delayed release formulations (Choi and Fenando, 2020).

#### Proposed Mechanism of Action

SSZ’s anti-inflammatory effects can be the result of either SSZ directly or its metabolites sulfapyridine and 5-aminosalicylic; the exact mechanism of action remains unknown. Several immunomodulatory mechanisms of action have been proposed for SSZ and its metabolites, including 1) the inhibition of NF-κB and thus its proinflammatory cascade and leukocyte accumulation (Wahl et al., 1998; Cronstein et al., 1999; Park et al., 2019), 2) the induction of caspase-8 induced macrophage apoptosis (Rodenburg et al., 2000), 3) the inhibition of RANKL (Lee et al., 2004), 4) the stimulation anti-inflammatory activity by facilitating adenosine accumulation via increased adenine conversion activity (Morabito et al., 1998), 5) B cell inhibition (Hirohata et al., 2002), and 6) the inhibition of the expression of several chemokines (Volin et al., 2002).

#### Clinical Evidence for Sulfasalazine

Though used for the clinical treatment of RA as early as 1948, SSZ did not gain ground as a recognized RA therapeutic treatment until many decades later (Suarez-Almazor et al., 2000b) after several controlled trials were conducted. A Cochrane systematic review of six placebo-controlled trials addressing SSZ activity as a monotherapeutic agent to treat RA found that SSZ is clinically effective as determined via tender swollen joint score, pain alleviation scores, and erythrocyte sedimentation rate (ESR) (Suarez-Almazor et al., 2000b) at a 6-month time point. Furthermore, patients in the SSZ groups were four times less likely to withdraw than patients receiving placebo (Suarez-Almazor et al., 2000b). Despite these benefits, the occurrence of adverse effects limits its use in a number of patients compared to other RA therapeutics (Suarez-Almazor et al., 2000b). SSZ as a combinatorial therapy with both MTX and HCQ is well tolerated and has been shown to be significantly more clinically effective in managing RA symptoms such as joint stiffness, joint swelling, pain, and ESR compared to MTX alone (O'Dell et al., 1996), SSZ and HCQ (O'Dell et al., 1996), MTX and HCQ (O'Dell et al., 2002), SSZ and MTX (O'Dell et al., 2002), and MTX and cyclosporin A (CSP) (O'Dell, 1998). This triple therapy regimen has been shown to have both comparable clinical outcomes and small radiographic differences when compared to combinatorial MTX and anti-TNF-α treatment after 2 years (Moreland et al., 2012). In addition to similar clinical efficacy, individuals found to be poor responders to MTX and anti-TNF-α combinatorial therapy have been successfully treated with triple therapy, and vice versa (O'Dell et al., 2013). Though determined to be as effective as MTX/TNFi treatment (Curtis et al., 2020), adherence to the triple therapy regimen was shown to be an issue during the two-year follow-up interval, with SSZ associated GI toxicity suggested to be the primary cause (Erhardt et al., 2019; Curtis et al., 2020).

### Hydroxychloroquine in RA

#### Overview

HCQ is an antimalarial medication first approved by the FDA in 1955 (Administration USFD, 2020). HCQ and its parent chemical chloroquine are 4-aminoquinolines, aromatic and planar in structure, with basic side chains that facilitate intracellular compartment accumulation – a process essential to their antimalarial mechanism of action (Schrezenmeier and Dorner, 2020). HCQ is enantiomeric and known to have stereoselective effects, but the widely prescribed formulation Plaquenil remains a racemic drug (Mok et al., 2005). Though HCQ has demonstrated immune-modulatory potential as a DMARD, it is not a panacea: HCQ has been shown to prevent bone destruction (Koduri et al., 2010), reduce atherosclerosis, protect against infections (Ruiz-Irastorza et al., 2009; Rempenault et al., 2018), possesses antithrombic (Sharma et al., 2016) capabilities, and yet has limited efficacy as monotherapy in severe RA. However, it is a safe and effective therapy for early and mild to moderate RA. Importantly, it serves as an effective component of combination therapy for aggressive RA (Tsakonas et al., 2000; Grigor et al., 2004; Moreland et al., 2012). The disparate effects from HCQ are believed to result from a variety of proposed mechanisms of action, with no singular mechanism resolutely accounting for its clinical efficacy (Schrezenmeier and Dorner, 2020).

#### Proposed Mechanisms of Action

Many mechanisms of action have been proposed for HCQ activity in RA and are thought to be related to disruption of lysosomal activity and its inhibition of antigen presentation and cytokine production. HCQ accumulates in the cellular lysosomes of B-cells, affecting lysosomal function by raising lysosomal pH, as seen *in vitro* (Circu et al., 2017; Mauthe et al., 2018). Proper lysosomal function enables antigen presentation and autophagy. As the hydrolytic activity of lysosomal enzymes is pH dependent, HCQ accumulation disrupts their function, subsequently attenuating MHC class II mediated autoantigen presentation, thus preventing antigen-induced T cell activation, expression of co-stimulatory molecules (such as CD154), and their subsequent immune response (Wu et al., 2017; Schrezenmeier and Dorner, 2020). There appear to be specific interactions within the lysosome responsible for this activity. One potential lysosomal target might be palmitoyl-protein thioesterase 1 (PPT1), an enzyme which cleaves lipids from proteins. PPT1 has been found to be upregulated in RA synovial tissue and is inhibited by HCQ *in vitro* (Rebecca et al., 2019 *)*. Perhaps PPT1 inhibitors may be a worthwhile area for future investigation (Ma et al., 2017).

Some anti-inflammatory aspects of HCQ have been attributed to reduced inflammatory cytokine production. These effects are due, at least in part, to inhibition of T cell activation, differentiation, and downstream T cell effector function resulting in reduced cytokine production. Additionally, HCQ interferes with TLR7 and 9 signaling by raising local endosomal pH (Ewald et al., 2008); and HCQ, like other antimalarials, may block nucleic acids from associating with TLR9 directly, as shown in colocalization assays using fluorescent spectroscopy (Kuznik et al., 2011). TLR signaling induces the production of cytokines, including IL-1, and disruption of this pathway reduces downstream TNF production and gene expression (Hjorton et al., 2018). HCQ has also been implicated in the reduction of other anti-inflammatory cytokines; *in vitro* studies have shown that HCQ can reduce the production of IL-1, IL-6, TNF, INFγ by mononuclear cells, and reduce TNF, INF⍺, IL-6, and CCL4 in plasmacytoid dendritic cells (pDC, an immune cell type linked to viral defense) and natural killer cell co-cultures (Wallace et al., 1993; Wallace et al., 1994).

#### Clinical Evidence for HCQ

Though HCQ is not recommended for use as a monotherapy for aggressive or established RA (Singh et al., 2015), there remains an important niche for this drug as an immune modulator with a low toxicity profile in RA treatment. Given the latter, rheumatologists frequently reach for its use in patients with contraindications to other more immune suppressive regimens. Additionally, in a Cochrane database systematic review, a statistically significant benefit was observed when HCQ was compared to placebo after 6 months of therapy, albeit with moderate effects (Suarez-Almazor et al., 2000a). Its use has been found to be most beneficial in early onset RA and in patients with mild to moderate disease activity (Tsakonas et al., 2000; Grigor et al., 2004). Currently, a U.S. placebo-controlled study entitled StopRA (Strategy for the Prevention of Onset of Clinically-Apparent RA) is evaluating whether HCQ can prevent or delay the onset of RA in individuals pre-determined to be at high risk of developing disease (based on family history and anti-CCP3 positivity ≥ 2 times the upper limit of normal, regardless of whether arthralgia is present) (Koffeman et al., 2009). Yet, as a monotherapy, HCQ failed to differentiate its efficacy from MTX and SSZ in more active disease despite being effective when used in a triple therapy regimen with these two other drugs (O'Dell et al., 2002; Ravindran and Alias, 2017). Clinically, HCQ is characterized by a long delay in the onset of action, which may result in withdrawal of this medication due to inefficacy (as reviewed in (Carmichael et al., 2002)). The slow onset of action can be attributed to its pharmacokinetics. It has a terminal half-life longer than 40 days; thus steady state is not reached until after 6 months of treatment (Tett et al., 1989). However, combination therapy with MTX and HCQ has been shown to be more potent than either medication used alone (Trnavský et al., 1993). Furthermore, it has emerged as an effective component of combination “triple therapy” for aggressive RA (Moreland et al., 2012).

### Prednisone in RA

#### Overview

Prednisone is a synthetic glucocorticoid (GC) derived from cortisone that has four to five times the anti-inflammatory potency of endogenous cortisone due to the existence of a double bond between its C1 and C2 atoms (Krasselt and Baerwald, 2014). Its robust activity as an anti-inflammatory and immunosuppressant has led to its extensive application as a therapeutic for acute and chronic immune conditions ranging from allergic response to chronic autoimmune diseases (Krasselt and Baerwald, 2014). If administered orally, prednisone is rapidly taken up through the small intestine for systemic circulation, where it has a plasma half-life of approximately 1 h (Krasselt and Baerwald, 2014). Prednisone is a biologically inert prodrug that is converted to its active form prednisolone via the hydrogenation of its C11 ketone group by liver metabolism. It is an important therapeutic to treat RA flares and quickly control disease, improve patients’ quality of life, and prolong and improve the efficacy of other csDMARDs (Krasselt and Baerwald, 2014). Side effects of prednisone such as hypertension, diabetes, myopathy, weight changes, and osteoporosis are largely dose dependent. However, low doses (usually considered <7.5 mg / day) can safely be used as disease modifying agents to treat RA with minimal side effects (Krasselt and Baerwald, 2014).

#### Mechanism of Action

Bioactive prednisolone is lipophilic, thus allowing the compound to passively diffuse through cell membranes (Krasselt and Baerwald, 2014). Once within cellular space, the drug associates with the cytosolic glucocorticoid receptor (cGCR), which triggers the release of receptor associated proteins and the translocation of prednisolone/cGCR to the nucleus, where it binds as a homodimer to GC responsive elements encoded in the cell’s DNA in a transactivation event that triggers an anti-inflammatory gene expression cascade (Krasselt and Baerwald, 2014). In addition to this genetic mechanism, GC/cGCR complex monomers are capable of interfering with the proinflammatory transcription factors NF-ĸB , activator protein-1 (AP-1) and nuclear factor for activated T cells (NF-AT), thus reducing the expression of major proinflammatory proteins IL-1, IL-6, and TNF-α (Krasselt and Baerwald, 2014).

#### Clinical Trials

Prednisone has been studied extensively in the clinical context of RA with beneficial results. The Utrecht study showed significant clinical benefit of 10 mg daily prednisone when administered as a monotherapy by inhibiting joint destruction, as determined via radiography (van Everdingen et al., 2002). The follow-up study to this clinical trial with two-years of prednisone treatment showed that even one-year after discontinuation of this drug, joint destruction inhibition was maintained (Jacobs et al., 2006). Another clinical trial of prednisone as an RA monotherapy showed significantly less people withdrawing from trial due to lack of efficacy compared to a placebo group (Pincus et al., 2009). Prednisone in combination with a DMARD has also been shown to achieve a higher remission rate, retard joint destruction, and initiate a more rapid clinical response compared to placebo controls (Wassenberg et al., 2005; Hafstrom et al., 2009; Bakker et al., 2012). In addition to direct therapeutic benefits, clinical studies have also suggested that prednisone may be able to prolong the survival time of csDMARD therapeutics for increased efficacy, as well as reduce the occurrence of csDMARD associated side effects (Malysheva et al., 2008).

### NSAIDs, COX-2, and Rheumatoid Arthritis

Historically considered a first-line treatment option for RA, nonsteroidal anti-inflammatory drugs (NSAIDs) have been replaced by conventional and biological DMARDs that provide joint protective effects. Though effective at relieving pain and inflammation associated with RA, chronic use of NSAIDs can result in cardiovascular and gastrointestinal (GI) toxicities such as acute coronary syndrome or stomach ulcers (Fitzgerald, 2004).

COX-2 inhibitors such as rofecoxib and celecoxib were developed in order to potentially prevent adverse GI side effects, keep up the anti-inflammatory properties, and provide additional pain relief.

In the late 90s and early 2000s, NSAID therapies for treating arthritis were limited and lacked effectiveness. Therefore, initially there was not a strong competitive NSAID market. However, the approval of Merck’s selective COX2 inhibitor rofecoxib (Vioxx) for RA in 1999 drove rapid industry wide innovation and lead to the release of Pfizer’s celecoxib in 2000. Both were shown to be effective in treating joint pain associated with osteoarthritis (OA) and RA and proved to reduce GI toxicity. While initially these products did not directly compete with each other, when rofecoxib was pulled from the market citing stroke and other cardiovascular risk concerns, celecoxib initially took a big hit in sales. Despite this, celecoxib was ultimately able to expand to the United States market.

While COX inhibitors play a minor role in RA treatment regimens, they play a larger role in the treatment of OA. For more information regarding MOA, please see previously published works (Krumholz et al., 2007; Ricciotti and FitzGerald, 2011; Zarghi and Arfaei, 2011).

### Cytokines in RA

#### Origins of Cytokine Response

CD4 T cells are known to play a key role in the pathogenesis of RA (Gay et al., 1993; Lundy et al., 2007; Plenge et al., 2007; Zikherman and Weiss, 2009). Yet, it remains unknown how arthritis-causing T cells initiate disease. Early events that lead to autoimmunity in RA prior to late manifestations of disease-specific immune dysregulation, such as overt joint inflammation, are currently not well-studied. However, it is believed that in the early pre-clinical phase of RA, there is a genetic component coupled with an environmental trigger prior to the onset of detectable systemic autoimmunity as reviewed by Deane and Holers (Deane and Holers, 2019). The strongest genetic association is with the MHC class II allele, HLA-DR4, supporting a role for antigen-presentation in disease (Plenge et al., 2007; del Junco et al., 1984). Polymorphisms in the HLA-DR4 allele can result in altered antigen binding to the MHC class II molecules present on the membranes of antigen presenting cell (APC) (Cruz-Tapias and Anaya, 2013). This leads to altered presentation of self-antigens to CD4 T cells resulting in their inappropriate activation and differentiation. Once these T cells, a subset of which likely recognize an intra-articular antigen (Ashouri et al., 2019), and other inflammatory immune cells enter the synovial microenvironment, arthritis is triggered. Environmental factors including smoking, stress, and hormonal changes (such as menopause) can trigger and enhance these genetic risk factors, though the precise mechanism is unclear. The reader is referred to this review (Edwards and Cooper, 2006) for more information regarding the hypotheses surrounding this topic.

**FIGURE 4:**
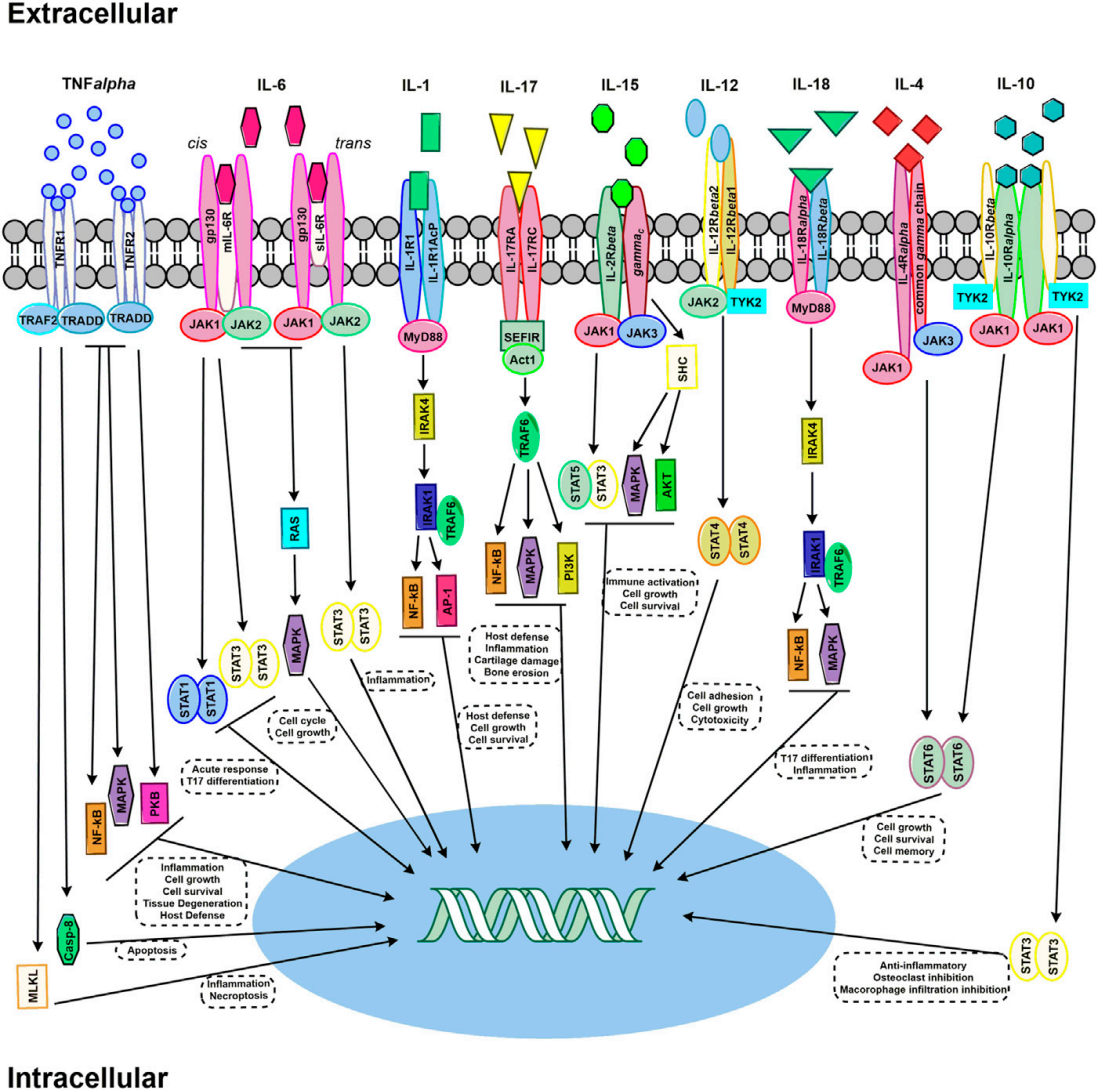
Select signaling pathways in RA. TNF-α signaling pathways required either TNFR1 or TNFR2 trimers. Signaling via TNFR1 pathway, upon TRADD binding without TNFR2, triggers cell death by either Casp-8 or MLKL. The recruiting of TRAF2 activates multiple signaling pathway cascade activation – including MAPK, NF-kB, and PKB. IL-6 signaling can occur through either mIL-6R classic signaling and of sIL-6R trans signaling. JAK activation occurs through both signaling mechanism and activating STAT and RAS/MAPK. IL-1 signaling through IL-1R1 via MyD88 which activates IRAK4 and subsequently IRAK1 bound to TRAF6 – leading to the activation of NFkB and AP1. IL-17 binds to an IL-17RA and IL-17RC receptor dimer. The SEFIR conserved signaling domain recruits Act1, which recruits TRAF6 and subsequently activates NF-kB, MAPK, and PI3K signaling pathways. IL-15 signaling can occur through JAK/STAT activation resulting in STAT3/STAT5 heterodimer formation, or activation through SHC which then results in activating MAPK and AKT. IL-12 signaling occurs through a heterodimer receptor consisting of IL-12Rβ1 and IL-12Rβ2 which activates JAK2 and TRK2 – leading to STAT4 dimer activation. IL-18 signaling results from the recruiting of MdD88 to the IL-18Rα and IL-18Rβ heterodimer, activating IRAK4 and thus TRAF6, which subsequently activates NF-kB and MAPK pathways. IL-4 signaling occurs the JAK/STAT activation via JAK1 and JAK3 binding to the IL-4Rα and common gamma-chain, respectively. IL-10 signal transduction results from both JAK1 binding to IL-10Rα and TYK2 binding to IL-10Rβ – which activates STAT3 in homodimer form.

During this pre-clinical phase of RA, as immune cells are activated and auto-antibodies become detectable, there is also an expansion of inflammation, marked by increasing levels of various cytokines and chemokines (Deane et al., 2010; Deane and Holers, 2019). These inflammatory pathways doubtlessly contribute to disease pathogenesis and select pathways that contribute to RA disease progression are outlined in Figure 4. Studying these final common mediators of disease have yielded breakthrough therapeutics.

#### The Role of Cytokines and Their Receptors in RA The Role of Targeted Cytokine Blockade in RA

##### TNF-α

By itself, the pro-inflammatory cytokine TNF-α is not inherently destructive. It is however, a potent chemo-attractant and the primary mediator in orchestrating an inflammatory response by promoting macrophage and lymphocyte proliferation, vasodilation, vascular permeability, and the expression of adhesion molecules by endothelial cells to aid in the extravasation of monocytes and neutrophils. In RA, TNF-α induces the proliferation of synovial lining cells and increases IL-1 synthesis. TNF-α acts synergistically alongside IL-1 to enhance the destructive effects of IL-1 resulting in increased bone and cartilage damage (Henderson and Pettipher, 1989). TNF-α binding to its receptors (TNFR1 and TNFR2) upregulates multiple signaling cascades within the target cell and triggers multiple pathways, such as the pro-inflammatory NF-κB pathway, RANKL signaling to induce osteoclast activation, the extra-signal regulated kinase (ERK) signaling pathway, and proapoptotic signaling that exacerbates inflammation (Farrugia and Baron, 2016).

Regulatory T cells (Tregs) are a subpopulation of T cells that are immunosuppressive in nature, responsible for the downregulation of effector T cells. Tregs by definition express the transcription factor forkhead box P3 (FoxP3), which acts as the master regulator in the function and development of Tregs (Fang et al., 2015). TNF-α is thought to suppress the anti-inflammatory actions of Tregs by downregulating FoxP3 expression (Farrugia and Baron, 2016), resulting in enhanced autoimmunity. Importantly, TNF-α has been shown to be a key cytokine in the initiation of RA, but further progression of the disease can occur independent of this cytokine (Mori et al., 1996).

There are two classes of membrane bound TNF-α receptors, TNF receptor 1 (TNFR1) and TNF receptor 2 (TNFR2). TNFR1 is present on most nucleated cells while TNFR2 expression is, for the most part, limited to immune cells (Choy and Panayi, 2001). TNFR1 mediates most of the host defense and inflammatory cellular signaling induced by TNF-α, while TNFR2 is thought to be essential in promoting T cell proliferation (Choy and Panayi, 2001).

RA patients have shown higher concentrations of soluble TNF receptors within the synovial fluid and serum, prolonging joint inflammation (Vasanthi et al., 2007). Upon binding with TNF-α, TNF receptors form a trimer, resulting in a conformational change of the cytoplasmic signaling domain. As a result, the inhibitory protein silencer of death domains (SODD) that associates intracellularly with TNFR1, is replaced with the adaptor protein TNFR1-associated death domain (TRADD). TRADD in turn recruits other proteins that mediate programmed cell death signaling and activates several pro-inflammatory pathways, including NF-kB, p38 MAP kinases, and apoptotic signaling (Chen and Goeddel, 2002).

#### IL-6

IL-6 is a pleiotropic cytokine produced by multiple cell types, including macrophages, monocytes, osteoblasts, bone marrow stromal cells, and fibroblasts (Akira et al., 1990; Pop et al., 2017). IL-6 plays a critical role in the pathogenesis of RA, as it is important for the maturation of B cells and thus, the production of auto-antibodies (Yoshida and Tanaka, 2014). IL-6 is also a direct stimulant of hepatocytes to promote synthesis of C-reactive protein (CRP) and is a critical regulator of CD4+ T cell differentiation and activation (Srirangan and Choy, 2010).

IL-6 plays a definitive and large role in the development and maintenance of RA symptoms. Serum taken from synovial fluid of RA patients demonstrated high expression of IL-6 (Madhok et al., 1993), and it is generally thought that IL-6 can promote joint damage and inflammation by acting on vascular endothelial growth factor (VEGF), an angiogenic mediator that promotes increases in vasculature and permeability (Nakahara et al., 2003). IL-6 plays an important role in the balance of Tregs and Th17 cells (Tanaka, 2013). IL-6 promotes Th17 cell differentiation through upregulation of the retinoid orphan receptor (ROR)γt, while inhibiting transforming growth factor-β-induced Treg differentiation (Korn et al., 2009). Th17 cells are critical for induction of tissue inflammation and destruction, which IL-6 exacerbates by offsetting the balance between Tregs and Th17 cells.

IL-6 induces cellular signaling by binding to a transmembrane IL-6 receptor (IL-6R) or a soluble form of the IL-6R (sIL-6R), which then associates and activates signal-transducing molecule gp130 through homodimerization (Taga et al., 1989). Gp130 recruits Janus kinases (JAKs), which then phosphorylate signal transducer and activator of transcription 1 (STAT1) and STAT3 to activate gene expression (Taga et al., 1989). Studies have associated IL-6’s pro-inflammatory responses with signaling through its soluble receptor, whereas signaling through its transmembrane IL-6R, IL-6’s canonical signaling pathway, is needed for its regenerative or anti-inflammatory properties(Rose-John, 2012).

#### IL-1

In addition to TNF-α and IL-6, IL-1 is a cytokine that is thought to play an important role in the pathogenesis of RA. IL-1 is produced predominantly by macrophages and monocytes as well as synovial fibroblasts, which is likely a critical source of IL-1 in RA (Deon et al., 2001). It acts as a potent chemoattractant, recruiting and activating lymphocytes and macrophages contributing to the inflammatory milieu. Inflammatory mediators induced by IL-1 signaling include IL-6, TNF-α, IL-8 and COX2 (Iwakura, 2002). These factors can lead to vasodilation and increased permeability of blood vessels, resulting in increased infiltration of inflammatory cells. Additionally, IL-1 can directly promote synovial cell growth, and activate synovial cells and osteoclasts to produce collagenases that induce bone and cartilage erosion (Mizel et al., 1981; Fontana et al., 1982; Saijo et al., 2002; Dayer, 2003).

IL-1A is an endogenous receptor antagonist secreted by activated monocytes and macrophages and can inhibit IL-1 signaling by binding to the IL-1 receptors (Gabay et al., 1997). In RA patients, IL-1A exists at significantly lower levels than IL-1 in the synovial fluid, likely permitting unrestrained IL-1 activity (Campion et al., 1996). Antibodies targeting the IL-1 receptor have been shown to reduce arthritis activity in animal models of RA, supporting the initial investigation of IL-1 as a therapeutic target in RA (Noack and Miossec, 2017). However, success in animal models has not translated to human studies (Buch et al., 2004; Burger et al., 2006)^,^.

There are two membrane bound classes of IL-1 receptors (IL-1R), types I and II. IL-1R type I is expressed across a variety of cells, including macrophages, lymphocytes, endothelial cells, fibroblasts, and synovial lining cells (Sims et al., 1993). IL-1R type II is expressed in low concentrations on monocytes, macrophages, B cells, and neutrophils (Sims et al., 1993). IL-1 binds both types of receptors with equal affinities. Signaling through type I is conducted through a long cytoplasmic tail in contrast with type II, which has a short cytoplasmic tail and is not functionally active (Dripps et al., 1991). The two-membrane bound IL-1 receptors, IL-1R1 and IL-1R2, have contrasting actions. IL-1R2 does not transmit signal and instead acts a decoy receptor that can inhibit IL-1 (Iwakura, 2002). Binding of IL-1 to IL-1R type I induces a conformational change in the receptor resulting in a heterotrimeric complex composed of the ligand, receptor, and a co-receptor. Formation of this complex brings together the intracellular Toll/IL-1 receptor (TIR) domains, leading to the recruitment of MYD88 and initiation of a pro-inflammatory cascade (Dinarello, 2019).

##### IL-17

IL-1 and IL-6 promote the differentiation of T cells into T helper 17 (Th17) cells, a subset of T effector cells that act as a source of IL-17 (Robert and Miossec, 2018). IL-17 receptors are expressed across most cells, but the key responsive types include non-immune cells such as epithelial and mesenchymal cells – the one implicated in RA pathogenesis being IL-17A (Robert and Miossec, 2018). Binding induces the expression of inflammatory genes, cytokines such as IL-6, and chemokines (CXCL1, CXCL2, CCL20). IL-17 is a potent amplifier of the inflammatory cascades induced by TNF-α, and is thought to upregulate the expression of TNFRII in synoviocytes (Zrioual et al., 2009), contributing to local inflammatory effects in the joints. Inhibiting IL-17 or its receptor using targeted antibodies reduces disease severity in rodent models of RA (Gaffen, 2009). Despite preclinical promise, human studies of IL-17 inhibition for the treatment of RA have, to date, been largely unsuccessful. This could be related to patient disease heterogeneity (variable expression of IL-17) and general IL-17 dysregulation in RA due to the many mediators that modulate its function (with both agonist or antagonist effects) (Robert and Miossec, 2018).

IL-17 binds to its cognate receptor IL-17R, to induce the synthesis of chemokines, which in turn recruit macrophages and neutrophils to the inflammatory location. IL-17 is a potent activator of the NF-kB and p38 MAP kinase signaling cascades, binding of the ligand to the receptor recruits E3 ubiquitin ligase TRAF6 (Monin and Gaffen, 2018). TRAF6 is an adaptor protein that indirectly binds to the IL-17R through intermediary protein Act1. Activation of TRAF6 leads to the attachment of ubiquitin chains on various targets, including inhibitor of nuclear factor kappa-B kinase subunit gamma (IKK-γ) which facilitates NF-κB activation.

TNF-α, IL-6, and IL-1 are major targets of pharmaceutical intervention ranging from small molecule drugs to more recent biologics. These therapies are discussed below.

#### Anti-TNF-α

As TNF-α is a potent pro-inflammatory cytokine that contributes to RA disease pathogenesis, it is a natural target for pharmacological intervention. TNF inhibitors (TNFi) were among the first biologics developed that successfully reduced disease activity in patients with RA that had failed csDMARD therapies, revolutionizing the treatment of RA (Keyser, 2011). For patients that have an incomplete response or have failed csDMARD, TNFi’s are often the first choice among biologic therapies for patients with RA as they have demonstrated high clinical efficacy in treating RA (Guo et al., 2018). Differences in formulation can have implications for disease-specific treatments, though anti-TNF therapies are almost all equally effective in treating RA, and effects are maximized in the presence of MTX (Ma and Xu, 2013).

Infliximab (IFX) was the first TNFi developed for RA and it acts to neutralize the biological activity of TNF-α by binding to all its forms (Lisman et al., 2002; Monaco et al., 2015). It is composed of a human antibody backbone with a mouse idiotype. Typical administration of this therapeutic is through an IV infusion, and IFX has been shown to be relatively safe for long term usage, though there are serious potential side effects seen with all anti-TNF-a agents, most important of which includes increased infection risk (Perdriger, 2009). A black box warning exists for patients with a known history of heart disease (namely congestive heart failure), as TNFi can contribute to exacerbation of disease in the setting of poorly compensated heart failure (Lisman et al., 2002) and infections (Perdriger, 2009). Though not a high risk, patients should be monitored for the occurrence of skin cancers may experience a slightly increased risk of lymphoma (Perdriger, 2009). In addition, patients receiving repeated IFX or biosimilar infusions are at risk of developing serum sickness (Vermeire et al., 2009; Scherlinger et al., 2017). Studies have shown decreased IL-1, IL-6, IL-8 and MCP-1 inflammatory mediators with IFX treatment (Braun and Kay, 2017).

Adalimumab (Ada) is a fully humanized anti-TNF-α monoclonal antibody typically delivered through a subcutaneous route. Ada controls RA disease activity more effectively when taken together with MTX, as the two have been shown to work synergistically (Breedveld et al., 2006). Studies have shown Ada to be a potent antirheumatic therapy, with many patients entering remission with improved disease scores (Machado et al., 2013).

Etanercept is composed of an immunoglobulin backbone and two soluble human TNF receptors. It is typically administered subcutaneously on a weekly basis. Etanercept is an effective anti-rheumatic agent, with remission rates of 21% as determined by the Disease Activity Score in 28 joints (DAS28) and 10% as determined by the Clinical Disease Activity Index (CDAI) (Hetland et al., 2010).

Golimumab is a human IgG1 kappa monoclonal antibody that binds to both the soluble and transmembrane bioactive forms of TNF-α. This therapy is administered subcutaneously every 4 weeks. Short term toxicity of this agent mirrors the other TNFi’s, however studies are needed to further investigate the long-term implications (Braun and Kay, 2017).

Certolizumab is a monotherapy of humanized antigen binding fragment of a monoclonal antibody bound to polyethylene glycol and is the only PEGylated anti-TNF-α biologic currently available to date (Goel and Stephens, 2010; Choy et al., 2012). Certolizumab is injected subcutaneously on a monthly basis, and though approved as a monotherapy by the FDA, it can also be used concomitantly with DMARDs for the treatment of severe RA (Goel and Stephens, 2010; Choy et al., 2012). In addition to having minimal side effects and in contrast to other TNFi’s, certolizumab is highly competitive in cases where pregnancy must be considered, given that it lacks the Fc region required for active transport across the placenta and therefore theoretically safer for use during pregnancy (Kaushik and Moots, 2005; Goel and Stephens, 2010).

#### Anti-IL-6

The pleiotropic cytokine IL-6 is thought to contribute to the differentiation of Th17 cells in human RA and targeting the IL-6R with clinically used humanized monoclonal antibodies leads to RA disease improvement (Fleischmann et al., 2013). Tocilizumab (TCZ) is an FDA approved humanized monoclonal antibody that targets the IL-6 receptor (IL-6R) on cell surfaces and in circulation for the treatment of RA. In RA, IL-6 can stimulate inflammation and increased bone resorption through the IL-6 receptors, making it an excellent target for pharmacological intervention. TCZ is available as an IV infusion or as a subcutaneous injection (Kaneko, 2013). There are several side effects associated with TCZ therapy, including increased risk of infection, increased retention of lipids, and the formation of life-threatening GI perforations in patients with GI diseases due to inhibition of gut wound healing activity (Kaneko, 2013; Gale et al., 2019). The LITHE phase III clinical study of TCZ found that RA patients treated with tocilizumab monotherapy had significantly better outcomes than MTX monotherapy in the context of structural joint damage as determined via the Genant-modified Total Sharp Score and the Health Assessment Questionnaire – Disability Index (Fleischmann et al., 2013). Tocilizumab is used for the treatment of moderate to severe RA disease activity in individuals who have either not responded to, or did not tolerate, more conventional treatments such as use of MTX (Jones et al., 2010; Fleischmann et al., 2013).

Sarilumab, another IL-6R inhibiting humanized monoclonal antibody approved by the FDA for the treatment of RA, demonstrated significant clinical improvement in American College of Rheumatology 20/50/70 response rates, Health Assessment Questionnaire, Disability Index, and Clinical Disease Activity Index remission in a phase three study when compared to adalimumab (Burmester et al., 2017), Administered via subcutaneous injection every two weeks, sarilumab shows high efficacy with only a slightly elevated risk of adverse events – the most common being injection site reactions and neutropenia (Burmester et al., 2017). Sarilumab is currently approved for the treatment of moderately to severely active RA in people who have either not responded to, or did not tolerate, more conventional treatments (McCarty and Robinson, 2018).

In addition to the two FDA approved IL-6R inhibitors, several other antibody-based biologics are currently undergoing clinical trials for the treatment of RA including olokizumab, levilimab, sirukumab, and clazakizumab (Tanaka and Martin Mola, 2014; Mease et al., 2016).

#### Anti-IL-1

Anakinra (Table 1), administered as a daily injectable, was the first IL-1 receptor antagonist on the market and FDA approved to treat RA (Mertens and Singh, 2009a). Targeting IL-1 for RA has been shown to reduce disease symptoms in some patients compared to placebo (Mertens and Singh, 2009b) and in combination with MTX compared to MTX alone (Cohen et al., 2002), however, the improvements were relatively modest in a large double-blind randomized control study, in contrast to the findings of TNF-a inhibitors (Bresnihan et al., 1998). This was thought to be, at least in part, due to anakinra’s short half-life (Campion et al., 1996). Additionally, a large excess of IL-1RA is required to block the effect of IL-1 (Arend et al., 1990; Dripps et al., 1991; Gabay et al., 1997). Side effects associated with this agent include injection site reactions, allergic reaction, and infection of the upper respiratory tract (Genovese et al., 2004; Mertens and Singh, 2009a). Interestingly, administration of this therapy showed improved cardiac contractility (England et al., 2018). Other inhibitors targeting the IL-1 pathway have been identified for potential applications in RA (e.g., rilonacept and an IL-1 converting enzyme inhibitor, pralnacasan), however, results to date have not demonstrated a robust clinically beneficial response (Terkeltaub et al., 2013).

#### The Role of Other Cytokines and Their Receptors in RA

Other cytokines (e.g., IL-15, IL-12, IL-18, IL-14, and IL-10) have been or are currently being explored in RA and RA therapy development. However, these targets have not been studied or utilized to the same extent as the above listed cytokines. For this reason, we are not covering them and their associated therapies in this review. To find out more about these cytokines please refer to the following literature: IL-15 (McInnes et al., 1996; McInnes and Liew, 1998; Ruchatz et al., 1998; Ogata et al., 1999; Ziolkowska et al., 2000; Waldmann, 2004); IL-12 and IL-18 (Presky et al., 1996; Joosten et al., 1997; Gracie et al., 1999; Dinarello et al., 2013); IL-4 and IL-10 (Cush et al., 1995; Joosten et al., 1997; Lubberts et al., 1998; Nelms et al., 1999; Shouval et al., 2014).

### JAK-STAT Signaling and Its Role in RA

The Janus kinase (JAK) – signal transducer and activator of transcription (STAT) pathway allows for the transferring of signals from cell membrane receptors to the nucleus (Seif et al., 2017). The JAK-STAT pathway plays a critical role in the development of the immune system and polarization of helper T cells (Seif et al., 2017). It mediates signaling by growth factors, chemokines and cytokines such as interleukins, interferons, hormones, and colony-stimulating factors via their cognate receptors (Fragoulis et al., 2019). These receptors associate with JAKs (Fragoulis et al., 2019). The JAK-STAT pathway plays a major role in the pathogenesis of RA and other immune-mediated diseases (Fragoulis et al., 2019). Pharmaceutical drug companies have developed therapeutics to target the JAK-STAT pathway for treatment of RA, primarily comprising of JAK inhibitors, also known as JAKi (Fragoulis et al., 2019).

Four different JAKs are found in humans: JAK1, JAK2, JAK3, and TYK2 (Seif et al., 2017). Each JAK includes four domains: N-terminal FERM domain, SH2 (Src Homology 2) domain, pseudokinase domain, and the conserved Protein Tyrosine Kinase (PTK) domain (Seif et al., 2017). The N-terminal FERM domain plays a large role in protein-protein interactions, and consists of three subdomains F1, F2, and F3. The SH2 domain mediates dimerization and activation of STATs (Seif et al., 2017). SH2 domains consist of nearly 100 amino acid residues, which bind to phosphotyrosine residues (Seif et al., 2017). The pseudokinase domain has no apparent catalytic functions but has regulatory roles (Seif et al., 2017). The fourth domain is the conserved PTK domain, which mediates phosphorylation of tyrosine residues located in downstream substrates (Seif et al., 2017). The conserved PTK domain at the C-terminus is made up of about 250–300 amino acid residues that form the catalytic region including the binding sites for substrates and ATP as the phosphate donor (Seif et al., 2017).

Seven different STATs exist in humans: STAT1, STAT2, STAT3, STAT4, STAT5A, STAT5B, and STAT6 (Seif et al., 2017). Each STAT includes four important domains: the unique N-terminus region, the coiled-coil domain, the DNA binding domain, and the trans-activation domain (Seif et al., 2017). The unique N-terminus region regulates STATs through the use of tetramer formation or tyrosine dephosphorylation. The coiled-coil domain plays a role in nuclear export and protein-protein interactions, both of which are critical for STATs to promote transcription (Seif et al., 2017). In order to bind to specific genes in the nucleus, STAT utilizes its DNA-binding domain. This domain recognizes the TTCN3-4GAAA sequence on the targeted gene and mediates sequence-specific binding. Lastly, the trans-activation domain is responsible for recruitment of specific proteins, specifically DNA polymerase II or histone deacetylases. The trans-activation domain is found in the C-terminus region and is made up of a conserved tyrosine amino acid residue (Seif et al., 2017).

One important feedback loop that is thought to be a major driver of RA pathogenesis is STAT3 (Krause et al., 2002; Ye et al., 2015). STAT3 is activated by a number of upstream cytokines including many from the IL-6 cytokine family, which associate with JAK1/2 and TYK2. STAT3 is also found to be constitutively active in RA synovial inflammation. One proposed mechanism of RA pathogenesis begins by either direct or indirect STAT3 activation by proinflammatory cytokines including IL-6, TNF-α and IL-1β. STAT3 activation then leads to increased expression of IL-6 family cytokines, inducing a positive feedback loop (Oike et al., 2017; Degboe et al., 2019). This group has also shown that genetic or pharmacological inhibition of STAT3 can decrease both inflammation and bone erosion in animal models. STAT3 also induces the cytokine Receptor Activator of NF-κB Ligand (RANKL). RANKL is a member of the TNF superfamily. It acts as the primary regulator of bone resorption and osteoclast formation (Papadaki et al., 2019). RANKL induces osteoclastogenesis, and differentiation of osteoclasts. Activation of RANKL is induced either directly or indirectly by IL-1β, IL-17, and TNF-α. Activated RANKL binds to Receptor Activator of NF-κB (RANK) of osteoclast precursors which then leads to bone erosion (Mori et al., 2011; Tanaka, 2019). In an animal model of RA in which TNF-α is overexpressed, absence of functional RANKL caused attenuation of the arthritic phenotype. Over expression of RANKL in the same mouse model accelerated onset of a severe RA phenotype (Papadaki et al., 2019). Additionally, the monoclonal antibody denosumab, targets RANKL and in clinical trials prevented bone erosion. However, the inflammation and other symptoms of RA remained, suggesting inhibition of RANKL is best used in conjunction with other anti-rheumatic therapies (Tanaka, 2019). The proposed delivery of drugs like MTX via mesenchymal stem cells proposed below could benefit from co-administration with an anti-RANKL medication to aid in cessation of bone erosion.

Development of effective STAT inhibitors can be both informed and complicated by the pathology of RA and the specific targeted STAT isoform or cell type. For example, STAT3 promotes cell survival and inflammation in lymphocytes and synovial fibroblasts, but in macrophages it is anti-inflammatory. This could present a cell type specific therapeutic target. STAT1 may also play a pathogenic or protective role in RA pathogenesis, depending on cell type and disease progression. However, in contrast to STAT3, STAT1 may increase expression of inflammatory genes in non-proliferating cells like macrophages but promote apoptosis and stop growth in lymphocytes and fibroblasts. Although development of STAT inhibitors may be challenging, they could be an important therapeutic target for RA moving forward (Oike et al., 2017). These examples illustrate the complexity of JAK/STAT signaling in RA, the potential pitfalls for drug development and the promise for more effective therapies targeting these pathways in RA and related autoimmune diseases.

JAK-STAT signaling begins with the binding of an extracellular ligand to its cognate receptor, which typically leads to conformational changes and tyrosine phosphorylations that result in the recruitment of JAKs to the intracellular signaling component of the receptors (Cronstein, 2005). (Harrison, 2012). Once JAKs associate with the receptor, they phosphorylate each other (Harrison, 2012). The JAKs further phosphorylate STATs, cytokine intracellular signaling domains of the receptors, as well as other downstream substrates (Harrison, 2012). STAT phosphorylation results in their activation and allows them to enter the nucleus where they are then able to induce transcription. STATs can bind as dimers as well as complex oligomers to target genes. In this way, the JAK-STAT pathway allows for control over transcription (Harrison, 2012). The aforementioned domains associated with both JAK and STAT play key roles during this pathway process and together allow for complex control over the movement of signals from the cellular membrane to the nucleus, and ultimately, for regulation over transcription to occur.

### JAK-STAT Inhibitors in RA

Tofacitinib was the first small molecule, reversible, non-selective JAKi FDA approved for the treatment of RA. It is slightly more selective for JAK1 and JAK3 compared to JAK2 and TYK2. The structure of tofacitinib and most JAKi’s mimics the adenosine portion of ATP and competitively binds to the ATP binding site in the tyrosine kinase domain. This binding inhibits the phosphorylation and activation of JAKs and the downstream phosphorylation and activation of STATs. As a result, cytokine production is decreased and the immune response dampened (Hodge et al., 2016). Tofacitinib was first approved for use in RA patients with inadequate response to MTX, the first line therapy for RA. Its approval had great impact in the advancement of RA therapeutics, as it identified a targeted, disease modifying immunomodulating therapeutic that can be used alone or in conjunction with DMARDs to benefit patients with poor response to traditional RA strategies (Kwok, 2014). Baricitinib, which was created based on the structure of tofacitinib, is a pan-selective JAK inhibitor as well, but with increased selectivity towards JAK1/2, moderately selective for TYK2 and much less so for JAK3 (Ghoreschi et al., 2011). Baricitinib demonstrated high efficacy and statistically significant improvements in patient joint pain compared to both placebo and adalimumab control groups in its phase III clinical evaluation (Keystone et al., 2017).

JAKi, as is the case with any immunomodulatory drug, can increase the risk of infections. Clinical trials for tofacitinib saw an increase in moderate infections like upper respiratory infections and viral gastroenteritis, and some cases of more serious infections like pneumonia and tuberculosis (Grigoropoulos et al., 2019; Itamiya et al., 2020). Most notably, the risk of varicella zoster virus reactivation seems to be increased compared to other immunomodulatory biologic agents (Itamiya et al., 2020). Other side effects of JAKi include cytopenias, anemias, and thrombocytopenia, as well as the potential for malignancy (Ghoreschi et al., 2011).This risk is thought to be due to JAK2 specific inhibition, as the cytokine receptors for erythropoietin and thrombopoietin signal through JAK2. Lipid profiles are also altered with JAKi treatment. For example, tofacitinib raises high density lipoprotein (HDL) and low-density lipoprotein (LDL) levels, but the mechanism is still unclear (Schwartz et al., 2016).

In an attempt to limit adverse events, more selective JAK inhibitors have been developed such as JAK3 selective inhibitors with promising efficacy and a concomitant reduction in side effects. One such JAK3 selective inhibitor was decernotinib, which made it through phase II trials for RA and has a five-fold greater selectivity for JAK3 compared to other JAKi’s (Gadina et al., 2016). JAK3 is only associated with Type I receptors of the common γ chain subgroup: IL-2, IL-4, IL-7, IL-9, IL-15, IL-21. These target T-cell proliferation and survival, memory, and regulatory cell function, as well as B-cell function and NK-cell activity (Conklyn et al., 2004; Soldevila et al., 2004; Chiossone et al., 2007; Robinette et al., 2018). JAK3 is primarily expressed in lymphocytes and within the hematopoietic system. Therefore, JAK3 selective inhibitors were thought to be promising drugs for RA as their effects would be limited to immune cells, and could mitigate other off-target side effects. The clinical trials for decernotinib showed promising efficacy and seemed to decrease anemia but had similar safety profiles and rates of infection compared to previous JAKi’s. Decernotinib development is currently no longer being actively pursued despite its positive clinical trial results due to decernotinib’s parent company, Vertex Pharmaceuticals, seeking opportunities for global development (Gadina et al., 2016; Westhovens, 2019).

JAK1 selective inhibitors are also an active area of RA drug development. The SELECT Phase III clinical trials evaluated the efficacy of the JAK1 inhibitor upadacitinib, now marketed as Rinvoq. SELECT-EARLY, SELECT-MONOTHERAPY, SELECT-COMPARE, SELECT-NEXT, and SELECT-BEYOND assessed upadacitinib with and without MTX or csDMARDS, and in total about 30% of patients achieved remission (Brooks, 2019). The development of upadacitinib illustrates some of the difficulties in designing JAK1 selective inhibitors. Upadacitinib was first described as ABT-494, a second-generation JAK1 selective inhibitor designed to exploit interactions outside of the ATP-binding site (Parmentier et al., 2018). ABT-494 was shown to be active against JAK1 (IC50: 47 nM) and JAK2 (IC50: 120 nM), but not JAK3(2304 nM) (Parmentier et al., 2018). However, it was found to be over 60-fold more selective for JAK1 over JAK2 when comparing IL-6 and Oncostatin M (OSM) induced STAT3 phosphorylation in TF-1 cells (a measure of JAK1 inhibition) over erythropoietin-induced STAT5 phosphorylation in UT-7 cells (a measure of JAK2 inhibition). It was presumed that this improved selectivity would abate potential off-target effects as intimated by similar IC50s for both JAK1 and JAK2. Though ultimately efficacious at both 15 mg and 30 mg, dose-dependent side effects emerged: In the SELECT-BEYOND trial, some patients receiving the highest dosages (30 mg/day) experienced a reduction in hemoglobin levels and subsequent anemia characteristic of JAK2 inhibition (Genovese et al., 2018). Thus, in this case efficacy was equal between lower and higher doses suggesting clinical usage requires careful balance between potency versus selectivity.

Filgotinib is currently being developed by the small molecule drug company Galapagos in collaboration with Gilead. Filgotinib, or GLPG0634, is a triazolopyridine JAK1-selective JAKi (Table 1) designed via a “screening cascade” to avoid JAK2 inhibition and subsequent hematopoiesis, a process which ultimately resulted in about 27-fold selectivity for JAK1 over JAK2 (Menet et al., 2014). Filgotinib is not yet on the market, but has undergone a multitude of clinical trials (Inc GS, 2019). The Phase II trials DARWIN I and II, demonstrated safety and efficacy with and without MTX for 12 weeks (Kavanaugh et al., 2017; Tarrant et al., 2020). A follow-through study named DARWIN III extended the treatment to 156 weeks and found 40% receiving monotherapy and 45% receiving combination therapy with MTX achieved ACR70, and 89.7% and 87.2% achieved ACR20 respectively (Campbell, 2019; Inc GS, 2020). The subsequent FINCH trials incorporate biologic therapies into the trials (Gallopagos, 2017). FINCH 1 examines filgotinib vs adalimumab vs placebo in patients that failed MTX. FINCH 2 examines filgotinib’s efficacy in patients that failed at least one biologic. FINCH 3 examines filgotinib as a first line therapy. The results of FINCH 2 concluded that a 12-week time course in filgotinib could improve ACR20, and that the most common adverse event was nasopharyngitis (the common cold) (Genovese et al., 2019), demonstrating value for JAK1 inhibitors in patients with poor response to adalimumab. In all, trials found that filgotinib could improve the RA disease score (ACR) and treatment response in patients who failed, or lacked a complete response to csDMARD therapies.

### T Cell Modulation in RA

As previously mentioned, CD4 T cells are known to play a role in RA disease pathogenesis. Their activation is an early event in the inflammatory process. Activation of the inflammatory cascade and production of inflammatory mediators results in inflammatory joint pain and damage. T cells require two signals for full activation: 1) signaling via the T cell antigen receptor (TCR), and 2) co-stimulatory signaling (e.g. through the T cell costimulatory receptor CD28). Interrupting T cell activation has therefore been explored as a therapeutic intervention for RA management (Maxwell and Singh, 2009).

Abatacept is a recombinant fusion protein biologic. It selectively inhibits T cell activation by binding costimulatory ligands CD80 and CD86, preventing their association with costimulatory receptor CD28, present on T cells (Maxwell and Singh, 2009; Blair and Deeks, 2017).

Abatacept has been highly studied in the clinical context of RA and was approved by the FDA in 2005 for the treatment of moderate to severe RA for adult patients who have not responded adequately to csDMARDs or TNF-α inhibitors. A Cochrane review of seven double blind randomized controlled clinical trials examining abatacept’s ability to treat RA demonstrated its high efficacy both as a monotherapy and in addition to other RA directed therapies (e.g., csDMARDs and biologics (Maxwell and Singh, 2009). The Cochrane review found groups treated with abatacept were significantly more likely to achieve an ACR50 response at one year, show significantly decreased disease activity, and demonstrate significantly improved physical functionality compared to placebo (Maxwell and Singh, 2009). Joint damage has also been determined to be significantly slowed in abatacept exposed groups compared to placebo as determined by radiographic progression at 12 months via a randomized control trial (Kremer et al., 2006). Cochrane review also determined that total adverse events and serious infections were greater in abatacept groups compared to placebo, and serious adverse events were only increased when given in addition to other biologics. Taken together, these studies have demonstrated that abatacept is effective and safe for the treatment of RA (Maxwell and Singh, 2009), and have successfully established it as an important therapeutic option for patients with RA who continue to experience disease activity despite csDMARDs and anti-TNFa therapies.

### B Cell Depletion and RA

The precise role of B cells in the pathogenesis RA is still somewhat controversial and not well understood. Several potential mechanisms of action have been proposed including B cell antigen presentation to autoreactive CD4+ T cells resulting in their activation, and B cell production and secretion of pathogenic autoantibodies (RF and anti-cyclic citrullinated peptide – CCP), proinflammatory cytokines and chemokines (Takemura et al., 2001; Dorner and Burmester, 2003; Shaw et al., 2003). Thus, B cell depletion has been used in the treatment of RA.

Rituximab (Shaw et al., 2003) is a chimeric monoclonal antibody reactive against human CD20, a B cell specific surface antigenic phosphoprotein, that acts to deplete B cell populations. Rituximab promotes B cell lysis or apoptosis as the result of recruiting macrophages, NK-cells, and monocytes via Fcγ receptor binding to B cell surface CD20 (Anderson et al., 1997; Clynes et al., 2000). In addition, CD20 binding by rituximab generates a membrane attack complex by complement dependent cytotoxicity induced by the complexing of rituximab with CD20 and C1q, resulting in B cell depletion (Reff et al., 1994; Weiner, 2010).

Initially approved for non-Hodgkin’s lymphoma (NHL), rituximab was first suggested as a potential RA therapeutic after RA remission was observed in patients treated for NHL that had coexisting RA (Shaw et al., 2003). After the results of a small scale exploratory open label study showed a positive impact (Edwards and Cambridge, 2001), a large randomized, controlled, double-blind study to evaluate rituximab efficacy in RA was conducted (Edwards et al., 2004), in which significantly more individuals reached ACR20 in all groups that received rituximab, either alone or in combination with either MTX or cyclophosphamide, compared to groups that received MTX alone at 24 and 48 weeks post either a single course or double infusion dosage (Edwards et al., 2004). Significantly more individuals achieved ACR20 and ACR50 in MTX and rituximab treated groups compared to MTX and placebo groups in the phase III REFLEX clinical trial (Keystone et al., 2012). In addition, rates of infection, adverse events, and serious adverse events remained comparable across all treatment groups – indicating no increased safety risk both initially and over time (Keystone et al., 2012). These results led to the FDA’s approval of rituximab in 2006 for individuals with moderate to severe RA whom demonstrated an inadequate response to TNF-α inhibitors. It is now readily available and not infrequently used in the management of RA.

### Dendritic Cell Vaccination as a Targeted Therapeutic for RA

There is currently no therapy on the market that has achieved antigen specific repression for controlling RA symptomology, and drug interventions have not induced long term remission or restoration of self-antigen immune tolerance – therefore lifelong treatments are typically required for RA (Thomas, 2013).

A critical component of autoimmunity is loss of tolerance to self-antigens. Tolerance mechanisms, both central and peripheral, exist to suppress self-reactive T-cells to maintain tolerant cell states, avoiding long terms effects of autoimmunity through modulation by NK-cells and T-cell populations (i.e. Tregs) (Thomas, 2013). The idea that autoreactive immune cells, which respond to and become activated by self-antigens (such as those related to RA), could potentially be eliminated via tolerizing immunotherapies is currently being explored. If successful, tolerizing immunotherapies could have the capacity to regulate and suppress autoreactive T cells without compromising off-target cell populations (Hilkens and Isaacs, 2013; Thomas, 2013). There are several approaches to stimulate this suppressive effect, including both *in situ* and *ex vivo* tolerization efforts. Although the original trend of tolerization research was to use direct dosing of proteins primarily through oral applications, recent endeavors have focused on *ex vivo* manipulation of dendritic cells, which are essential for the induction and maintenance of immune cell tolerance (Kavanaugh et al., 2003; Hilkens and Isaacs, 2013).

#### Oral Tolerization

Attempts to stimulate this effect *in situ* through oral/mucosal or skin-based antigen application have been pursued with a variety of peptides, but have had varied success in clinical trials in the context of autoimmune disease, despite ease of introduction, the peptides being incredibly well tolerized, and positive outcomes in allergy desensitization.

Oral tolerization of collagen type II demonstrated early clinical efficacy in a three month-long double-blind clinical trial involving 60 patients with severe RA (Trentham et al., 1993). After being fed chicken type II collagen daily over the experimental period, patients experienced a decrease in joint sensitivity and swelling (Trentham et al., 1993).

In another phase II clinical trial, oral administration of JP1, a bacterial heat shock peptide with high sequence identity to the shared epitope sequence of a pathogenic RA autoimmune inflammatory protein encoded by human leukocyte antigen class II alleles (HLA-DR SE) in RA, resulted in a qualitative change from proinflammatory to a tolerogenic phenotype, and, in post hoc analysis, suggested a potentially synergistic effect when combined with HCQ in (Koffeman et al., 2009).

Though the results of these trials were quite promising, as measurable induction of regulatory populations and general immune deviations towards less pathogenic cytokine secretion were documented, significant long-term clinical efficacy was not achieved and motivates further study (Kavanaugh et al., 2003; Koffeman et al., 2009; Thomas, 2013). Several reasons have been cited for these therapies’ limited clinical benefits including narrow antigen dose window, the varied capacity of Tregs to suppress self-antigen cytokine production, the interference of microflora at mucosal interfaces with antigen presentation, and that activated autoimmune T cells are more resistant to tolerizing mechanisms than naïve T cells (Thomas, 2013).

#### *Ex-vivo* Tolerization

To usurp *in situ* limitations, dendritic cells and their precursors can be isolated from peripheral blood or removed from tissues, such as bone, for manipulation *in vitro* so autoantigens can be presented with higher fidelity. *Ex vivo* manipulation appears to increase the capability of dendritic cells to eliminate auto-antigen-specific T cells and activate Treg cells before being reintroduced *in vivo* (Harry et al., 2010; Thomas, 2013). This method, due to promising *in vitro* and preclinical model results, has led to the development of several clinical tolerogenic dendritic cell preparatory protocols and the execution of a clinical trial focusing on this as a cell therapy (Ahmed and Bae, 2016).

Cells used in this capacity for clinical application have general preparation guidelines spelled out by Good Manufacturing Practice of harvesting and cell culturing with several drugs and/or factors that support directed suppression against RA (Harry et al., 2010).

The first clinical trial of tolerogenic dendritic cells for the treatment of RA was rheumavax – which showed highly promising safety and efficacy data in patients with early RA. The tolerogenic T cells were isolated and treated with a modified NF-κB inhibitor and exposed to 4 RA associated peptide antigens before being reintroduced to a patient with RA (Benham et al., 2015). After a single injection, there were measurable increases in Treg cells and decreases in pathogenic T cells.

Although the challenges facing this branch of therapeutics are the same as any autologous cell therapy, such as standardization protocols for personalized medicine treatments and designing adequate controls, these results are very promising in the continued development of autologous cell therapies for targeted autoimmune repression (Thomas, 2013).

### Nanoparticle Drug Delivery Systems for RA

Nanoparticles are ultrafine colloidal particles with diameters in the size range of 1–1000 nm (Mohanraj and Chen, 2006). Nanoparticles and other nanomaterials have a wide range of applications in medicine both in diagnostics, such as magnetic resonance imaging (MRI) contrast agents, and in therapeutics as drug delivery vehicles (Fang and Zhang, 2010). Nanoparticles offer several benefits for delivery of drugs to specific sites at optimal rates and doses. Nanoparticles can help optimize the pharmacokinetics of drugs by enabling spatially and temporally controlled drug delivery (Owens et al., 2006). Nanoparticles can be used for targeting specific cell types and can improve drug circulation time by protecting drugs from degradation and by allowing sustained release.

The chemical and physical properties of nanoparticles such as material, size, and surface coatings greatly affect their potential biomedical applications (Stevenson et al., 2011). Nanoparticles can be produced from several materials including metals, polymers, silica, phospholipid bilayers, liposomes, and inorganic dyes. Nanoparticle size can be controlled through the fabrication process and can be used to control uptake by cells, drug loading, and drug release rates (Mohanraj and Chen, 2006). Nanoparticle surface coating is important for biocompatibility, can be used for cell targeting, and is important for controlling the clearance of nanoparticles. Nanoparticle coatings with functionalized surface groups permit the conjugation of drugs as well as the targeting of cells - such as in folate acid coated particles that target activated macrophages expressing folate receptor β (FRβ) (Nogueira et al., 2016).

Nanoparticles are being investigated as drug carriers for treatment of RA and have been combined with many of the common therapeutics including NSAIDs, glucocorticoids (GCs), csDMARDs, and biologics (Pham, 2011).

NSAIDs were once widely used in RA due to their analgesic and anti-inflammatory effects. However, variable differences in efficacy and side effects at high doses have limited the long-term use of NSAIDs in RA. The combination of NSAID with nanoparticles is being explored as a possible solution to overcome these limitations (Pham, 2011). Polymeric nanocapsules were prepared with a polysorbate coating, which prolongs circulation time by delaying the binding of plasma proteins (Bernardi et al., 2009). The nanocapsules were loaded with indomethacin and exhibited potent anti-inflammatory effects in an adjuvant-induced model of chronic arthritis, as evidenced by markedly depressed serum levels of pro-inflammatory cytokines TNF-α and IL-6 and enhanced levels of the anti-inflammatory cytokine IL-10 (Bernardi et al., 2009). An iron/ethylcellulose (core/shell) nanoparticle loaded with the NSAID diclofenac allowed for high drug loading capacity and prolonged drug release while also permitting targeting of diclofenac to inflamed joints under the guidance of an external magnetic field (Arias et al., 2009).

GCs are fast acting anti-inflammatory compounds but are rapidly cleared following systemic administration resulting in the need for high and frequent dosing, which increases the risk of adverse effects (Pham, 2011). Nanoparticles are being used for controlled release of GCs to improve circulation time and reduce dosing frequencies. PEG-liposomes (∼100 nm in size) encapsulating the GC prednisolone remained in circulation with a half-life of 50 h. A single systemic administration of this preparation led to complete reversal of paw inflammation within 2 days of injection in mice with adjuvant-induced arthritis (AIA). This effect lasted 2 weeks and therapeutic activity was observed at GC doses 100-fold lower than that of unencapsulated GC (Metselaar et al., 2003). Linear cyclodextrin polymer (CDP) nanoparticles conjugated with α-methylprednisolone (MP) were shown to be effective in reducing arthritis in murine CIA at doses up to 100-fold lower than free MP using weekly injections (Hwang et al., 2008).

NPs made from the biocompatible polymer poly(lactic-co-glycolic acid) (PLGA) and targeted with the arginine–glycine–aspartate (RGD) peptide sequence. RGD is a sequence of extracellular matrix and other proteins that binds to αvβ3 integrin and other αv- integrins. Such NPs have been used for STAT1 siRNA delivery leading to regress of RA in mouse models by silencing STAT1 leading to an increase in expression of IL-10 mRNA (Scheinman et al., 2011). In one study, tocilizumab-loaded hyaluronate-gold nanoparticles (HA-AuNP/TCZ) targeted with a monoclonal antibody against IL-6 were tested in CIA mice showing an antiangiogenic effect (Lee et al., 2014).

Nanoparticles in combination with various drugs have been investigated for treatment of RA with drug-particle combinations consistently showing marked improvement over free drug alone. By carefully selecting the chemical and physical properties of nanoparticles it is possible to create an efficient drug delivery system for a selected drug of choice. The use of nanoparticles has great potential in RA when combined with previous drug therapies or for combination with as of yet unexplored drug therapies.

### Prospective Novel RA Therapy: Mesenchymal Stem Cell Drug Delivery

Currently, csDMARDs, such as MTX, and biologic therapies are the mainstay of RA treatment. However, these treatments can have severe side effects including nausea, fatigue, and headache. Localized release via a drug delivery vehicle could provide greater spatiotemporal control over the drug to maximize efficacy at the inflamed joint while minimizing unwanted dose-dependent off-target side effects. This therapy would rely on a robust delivery method to the inflamed joint and the use of a biomaterial that can release the drug over several weeks. We propose to utilize mesenchymal stem cells (MSCs) as a “shuttle” to the inflamed joint to deliver polymeric poly (lactic-co-glycolic acid) nanoparticles loaded with MTX.

#### MSCs as a Drug Delivery Shuttle

MSCs are multipotent cells that can be harvested from many different tissues such as bone marrow, adipose tissue, or umbilical cord tissue. These cells alone have the potential to treat inflammatory diseases through their anti-inflammatory properties including exosome, cytokine, and growth factor secretion (Ren et al., 2012). Furthermore, MSCs have been observed to migrate to sites of inflammation after injection (Rustad and Gurtner, 2012). This unique property has strong potential to be utilized as a “shuttle” for this drug delivery approach. An anti-inflammatory drug (e.g., MTX) could be loaded into a slow-release nanoparticle formulation. These nanoparticles can be conjugated onto the surface of the MSCs before injection. Furthermore, the MSCs could further promote immunosuppression through their excreted growth factors and cytokines that may synergize with the drug. A similar delivery system has been developed by Xia et al. although not for use in RA (Xia et al., 2019). The MSCs, with their drug “backpacks” can home to sites of joint inflammation to deliver the biomaterial drug cargo. The released biomaterial could then act as a slow-releasing drug depot within the joint.

#### Poly (Lactic-Co-Glycolic Acid) Nanoparticles as a Biomaterial Drug Carrier

An ideal material for delivery to the inflamed RA-affected joint must have tunable release kinetics and strong biocompatibility. We propose to use PLGA, a widely used FDA-approved biomaterial for this arthritis therapy. PLGA can be formulated into nanoparticles through a simple oil-in-water emulsion with loaded drugs. The drug release kinetics can be tunable from the scale of days to months based on the lactic/glycolic acid ratio and molecular weight of the polymer (Amann et al., 2010). The nanoparticles typically are cleared by the liver and kidney, with the material itself being highly biodegradable with no toxic effects (Rempenault et al., 2018). Recently, it has also been discovered that PLGA has latent immunosuppressive effects, likely due to release of lactic acid upon degradation (Allen et al., 2018). This property could further reduce inflammation at the target joint site.

#### Site-Specific Cleavable Linker for Targeted Release

To create a long-lasting drug release depot, the nanoparticles must stay at the site of inflammation for weeks to months. However, previous work has shown that MSCs only survive in the body for 24 hr after intravenous injection (Eggenhofer et al., 2012). To circumvent this issue, we propose a cleavable peptide linker could be used to tether the PLGA nanoparticles to the shuttle MSCs. Matrix metalloprotease enzymes (MMPs) are found in abundance at the site of RA-affected joints (Rengel et al., 2007). The cleavable linker will contain a GPVGLIGK peptide designed by Zhang et al. (Zhang et al., 2016) for MMP-2 and MMP-9 both found in inflamed RA synovial fluid. This therapy could allow injected MSCs to shuttle MTX-loaded PLGA nanoparticles to the inflamed joints, where the peptide linker would be cleaved by disease-specific MMPs at the target delivery site thereby release the drug releasing nanoparticles.

## Conclusion

Treatment for RA has made many advances in the decades since the approval of MTX for RA in 1988. While MTX to date remains the first line treatment for patients with RA, a new class of advanced biologics in the form of antibody therapies has made great leaps forward in precisely targeting a myriad of pathways to robustly alleviate joint inflammation. Additionally, the next generation of JAK inhibitors is gaining FDA approval, offering even more options for patients to control RA symptoms. Despite these advances, many patients still have incomplete control of their RA or face side effects that they cannot easily live with. As our understanding of RA grows and we appreciate the mechanisms that cause individual variance of RA symptoms and treatment effects in patients, RA therapies will continue to become more precise, either through improved administration methods or with individualized targeted therapies. This precision medicine approach to rheumatic diseases such RA and other autoimmune diseases may one day resemble tailored therapy regimes now common in the field of oncology, achieving a patient-specific standard of care to yield optimized efficacy with minimal occurrence of side effects.
